# Identification of Active Markers of Chinese Formula Yupingfeng San by Network Pharmacology and HPLC-Q-TOF–MS/MS Analysis in Experimental Allergic Rhinitis Models of Mice and Isolated Basophilic Leukemia Cell Line RBL-2H3

**DOI:** 10.3390/ph18040540

**Published:** 2025-04-07

**Authors:** Xinqi Li, Caining Zhao, Jin Qi

**Affiliations:** Research Center for Traceability and Standardization of TCMs, School of Traditional Chinese Pharmacy, China Pharmaceutical University, Nanjing 211198, China; 3120020134@stu.cpu.edu.cn (X.L.); 17312307045@163.com (C.Z.)

**Keywords:** Yupingfeng San, allergic rhinitis, prototype component, network pharmacology, molecular docking

## Abstract

**Background:** Yupingfeng San (YPFS) is a classic formula for treating allergic rhinitis (AR), which is composed of *Astragalus mongholicus* Bunge (AST), *Atractylodes macrocephala* Koidz (AMR), and *Saposhni-kovia divaricata* (Turcz.) Schischk (SR) at a ratio of 3:1:1. However, the potential bioactive components of YPFS relevant to AR treatment are currently unknown. **Methods:** This study combined in vivo chemical profiling, network pharmacology, and experimental validation to identify the substances in YPFS that are active against AR. **Results:** Firstly, 98 compounds in YPFS were identified using high-performance liquid chromatography–quadrupole time-of-flight mass spectrometry (HPLC-Q-TOF-MS/MS) with the assistance of Global Natural Products Social (GNPS) molecular networking. Then, 42 prototype components and 57 metabolites were detected in the plasma, urine, and feces of mice with AR. A network pharmacological analysis based on 42 in vivo prototypical components was also conducted to screen 15 key components and 10 core targets, and 6 key components were further selected through molecular docking. Finally, the four key active components (cimifugin, wogonin, formononetin, and atractylenolide I) were revealed to be the main ingredients of YPFS through validation (in vitro and in vivo). **Conclusions:** This is the first systematic study of the components of YPFS in AR mice, laying the foundation for elucidating the overall material basis of this formulation. This study provides rich basic data for further pharmacological and mechanistic studies on YPFS.

## 1. Introduction

Allergic rhinitis (AR), a common allergic condition, is triggered by immunoglobulin E (IgE) in response to allergens such as pollen [1,2]. As per the 2020 publication of the *White Book* by the World Allergy Organization, the worldwide occurrence of allergic disorders varies, ranging from 10 to 40% of the population, with 400 million cases of AR, urticaria, and anaphylactic shock [3,4]. In China, epidemiological studies showed a high prevalence of AR, affecting approximately 250 million people (17.6%) with a continuous upward trend [2]. Therefore, developing new medications for treating AR is of great practical importance.

Yupingfeng San (YPFS) has demonstrated remarkable effectiveness in treating AR, authored *Danxixinfa* by Zhu Zhenheng during the Yuan Dynasty, and has demonstrated remarkable effectiveness in treating AR. It is composed of three herbs—*Astragalus mongholicus* Bunge (AST), *Atractylodes macrocephala* Koidz (AMR), and *Saposhnikovia divaricata* (Turcz.) Schischk (SR)—at a ratio of 3:1:1 [5]. The chemical components of YPFS are diverse, encompassing flavonoids, saponins, coumarins, chromones, and lactones [5]. However, the material basis on which YPFS treats AR remains unclear, and its complex metabolism in the body complicates the issue [6]. Therefore, it is imperative to employ modern scientific methods to elucidate the material basis and mechanism by which YPFS treats AR.

Most prior studies have primarily focused on validating the effectiveness of YPFS in mitigating inflammatory factors in AR [7,8]. Some reports have also identified the absorbed components of YPFS in the blood of rats [9]. However, the prototype components of YPFS in AR models have not been reported. Numerous studies have demonstrated that the absorption and metabolism of traditional Chinese medicine (TCM) differ significantly in pathological conditions compared to natural and healthy states [10,11]. Hence, it is important to identify the essential components of YPFS for managing AR.

This study introduced a systematic approach integrating high-performance liquid chromatography–quadrupole time-of-flight mass spectrometry (HPLC–ESI-Q-TOF-MS/MS) and Global Natural Product Social Molecular Networking (GNPS) to comprehensively identify the chemical components of YPFS. In vivo, we identified the prototype components and metabolites in the plasma, urine, and feces of AR mice after the oral intake of YPFS using mass spectrometry. Then, we constructed a network of the interactions between the prototype components of YPFS and AR-related targets to identify the key components and core targets and predict the potential pathways involved in the regulation of AR. The relationships between the core components and targets were further evaluated through molecular docking, and the components’s anti-AR effects were validated in RBL-2H3 cells and AR mice. This study serves as a foundation for understanding the therapeutic potential of YPFS in AR and provides a scientific basis for screening active ingredients in other medications.

## 2. Results

### 2.1. Optimization of Chromatography Conditions

The parameters were optimized to increase the separation of YPFS and improve the peak shape and number of peaks. As depicted in Figure S1, the findings revealed that a sample size of 5 mg, flow rate of 1 mL/min, column temperature of 30 °C, and wavelength of 254 nm were the most conducive to the effective separation and identification of the compounds within YPFS. The RSD values of all components were less than 3%, indicating that the method exhibits good repeatability, precision, and stability (Table S1–S3).

### 2.2. Chemical Profile of YPFS

**Figure 1:**
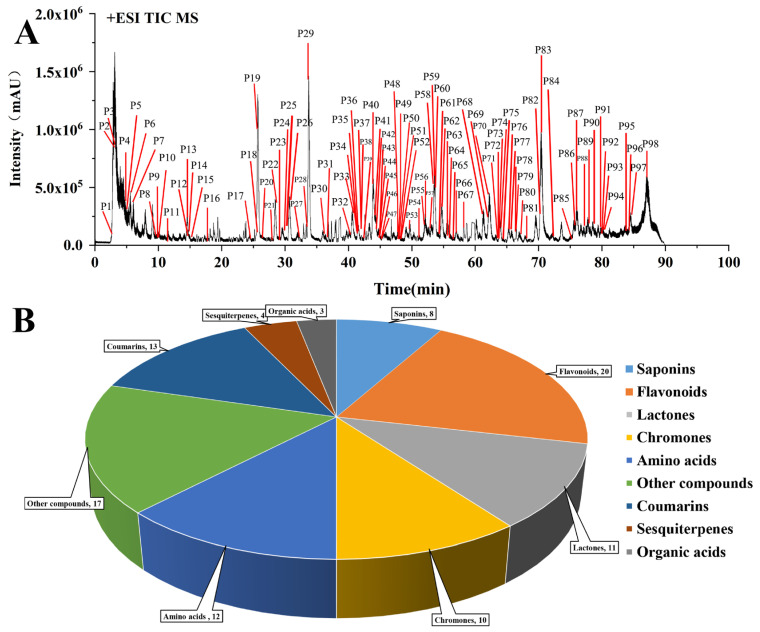
A total of 98 chemical components of YPFS identified through manual identification and GNPS in positive ion mode. (**A**) Base peak chromatograms of YPFS obtained using HPLC–Q-TOF-MS in positive modes. (**B**) Types of chemical compositions in YPFS.

The chemical profile of YPFS was systematically analyzed using manual identification and GNPS methods. A total of 98 compounds (Figure 1A and Table S4) were identified, comprising 20 flavonoids, 8 saponins, 11 lactones, 4 sesquiterpenoids, 10 chromones, 13 coumarins, 12 amino acids, 3 organic acids, and 17 other compounds (Figure 1B).

The molecular network of YPFS was established using GNPS for the analysis of natural products [12]. As depicted in Figure S2, the generated molecular network comprised 39 clusters (each cluster containing ≥ 2 nodes), totaling 388 nodes, with 89 feature components predicted.

**Figure 2:**
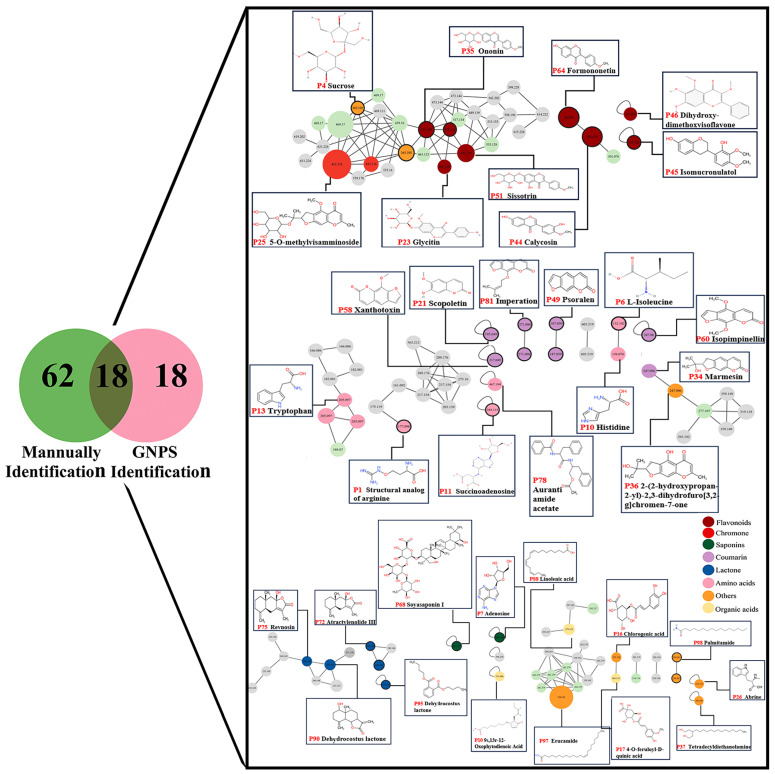
The structures of common components identified manually and using GNPS. The node color indicates the type of compound (red: flavonoids; green: saponins; purple: coumarin; blue: lactone; pink: amino acids; orange: others; pale yellow: organic acids).

The data were subject to the rigorous deduplication and deletion of the endogenous components based on comparisons with literature, chemical compound databases, and MS data [5,13]. In total, 36 potential chemical components of YPFS were obtained. Their chemical structures are shown in Figure 2, including six flavonoids, seven amino acids, two saponins, three organic acids, one chromone, six coumarins, four lactones, and seven other compounds. Notably, 18 components were consistent with previous manual identification predictions, further validating the methodology. This analytical method expedited the identification of chemical constituents within intricate natural product matrices.

Using the degree of similarity in the MS/MS spectra of individual compounds, which ranges from 0 to 1 in cosine similarity, compounds could be grouped into clusters within a molecular network. This clustering method enabled the rapid classification of components [14]. As illustrated in Figure 2 and Figure S2, the node associated with the ion at *m*/*z* 431.134 corresponded to the molecular formula C_22_H_22_O_9_. Analysis of the MS/MS spectrum revealed fragment ions with mass-to-charge ratios of 269, 254, and 137, which are indicative of glycoside ligands, methyl groups, and compounds formed through the retro-Diels–Alder reaction (RDA). By cross-referencing the retention time and MS data with existing databases and scientific literature, the compound was conclusively identified as ononin [15]. The node of the ion at *m*/*z* 447.129 was adjacent to the node of ononin, suggesting a high degree of MS/MS spectral similarity with an excimer ion difference of 16 Da. Based on literature reports [16,17], this node had been identified as sissotrin. Similarly, other nodes were identified through this process.

### 2.3. Characterization of Compounds

Compounds with similar structures exhibited comparable cleavage patterns. Therefore, it is imperative to analyze the cleavage rules of the prototype components in order to accurately identify the metabolic components of YPFS in vivo. In this study, we analyzed several typical compounds present in YPFS.

#### 2.3.1. Flavonoids

**Figure 3:**
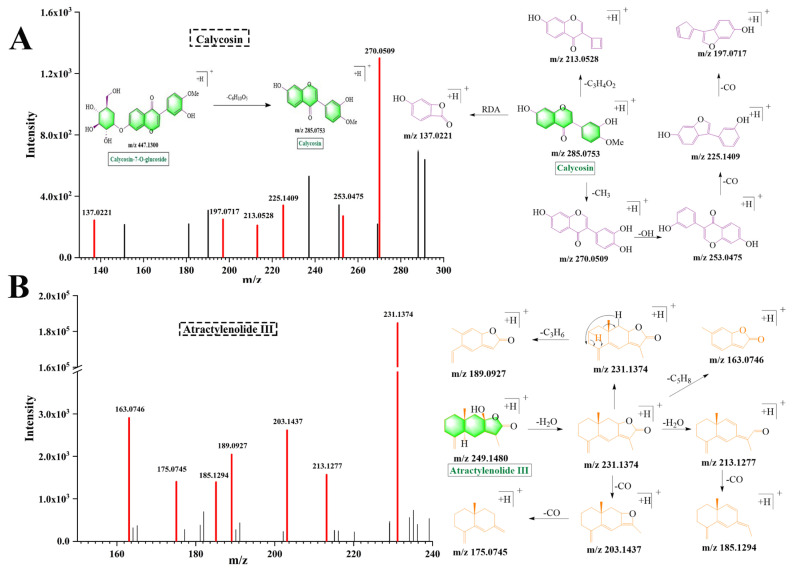
Chemical fragmentation pathways of chemical components in YPFS. (**A**) calycosin; (**B**) atractylenolide III.

Initially, 19 flavonoids were identified in YPFS, primarily originating from AST. These flavonoids were identified based on characteristic neutral losses of CO (28 Da), CO_2_ (44 Da), and CH_3_ (15 Da). Calycosin (Figure 3A) and ononin (Figure S3A) exhibited the same cleavage pathway [18,19]. The cleavage rules of flavonoids were represented by the example of calycosin. Calycosin exhibited an [M + H]^+^ ion at *m*/*z* 285.0753, which was formed by the loss of glucose (C_6_H_10_O_5_, 162 Da) from calycosin-7-O-glucoside. The fragment ion at *m*/*z* 285.0753 [M + H]^+^ further generated the fragment ions at *m*/*z* 270.0509, *m*/*z* 213.0528 and *m*/*z* 137.0221 through losing CH_3_ (15 Da), C_3_H_4_O_2_ (72 Da) and RDA. Additionally, fragment ions at *m*/*z* 225.1409 and 197.0717 were produced through the sequential loss of CO (28 Da) from the fragment ion at *m*/*z* 270.0509.

#### 2.3.2. Lactones

Analogously, 12 lactones were identified in YPFS, primarily originating from AMR. The cleavage rules of lactones were represented by the example of atractylenolide III (Figure 3B). Atractylenolide III exhibited an [M + H]^+^ ion at *m*/*z* 249.1480. Fragment ions at *m*/*z* 231.1374 and *m*/*z* 213.1277 were generated through the sequential loss of H_2_O (18 Da) from atractylenolide III. The fragment ion at *m*/*z* 213.1277 underwent further loss of CO (28 Da) to form the fragment ion at *m*/*z* 185.1294. Additionally, fragment ions at *m*/*z* 203.1437 and *m*/*z* 175.0745 were formed by the sequential removal of CO (28 Da) from the fragment ion at *m*/*z* 231.1374. Lastly, the fragment ions at *m*/*z* 163.0746 and *m*/*z* 189.0927 were generated by the loss of C_5_H_8_ (68 Da) and C_3_H_6_ (42 Da) groups from the fragment ion at *m*/*z* 231.1374.

#### 2.3.3. Coumarins

A total of 12 coumarins were identified in YPFS, primarily originating from SR. These coumarins could be classified into two distinct categories, furanocoumarins and coumestrol, based on the structure of their parent nuclei. The characteristic fragment ions of coumarins, specifically those at *m*/*z* 131, 143, and 159, were detected in positive mode [20]. Psoralen (Figure S3B) and imperatorin (Figure S4A) shared the same cleavage pathway. Taking imperatorin as an example, the cleavage rule of coumarins is described below. Imperatorin exhibited an [M + H]^+^ ion at *m*/*z* 271.0956, whose cleavage rule involved the continuous loss of CO (28 Da). The fragment ion at *m*/*z* 203.0330 was obtained through the loss of the C_5_H_8_ (68 Da) group. There were three possible fragmentation pathways: Firstly, fragment ions at *m*/*z* 175.0361, 147.0458, and 119.0856 were produced by the continuous loss of CO (28 Da) from the fragment ion at *m*/*z* 203.0330. Alternatively, the fragment ion at *m*/*z* 203.0330 could lose CO_2_ (44 Da) to form the fragment ion at *m*/*z* 159.0965, which subsequently lost CO (28 Da) to form the fragment ion at *m*/*z* 131.1069. Additionally, the fragment ion at *m*/*z* 203.0330 could lose H_2_O (18 Da) to obtain the fragment ion at *m*/*z* 185.0900. Fragment ions at *m*/*z* 157.0509 and 129.0552 were generated by the continuous lost of CO (28 Da) from the fragment ion at *m*/*z* 185.0900 [21].

#### 2.3.4. Chromones

Currently, the chemical constituents characterized by SR primarily comprise two distinct classes: dihydrofuran chromogen ketones and dihydropyran chromogen ketones. Dihydrofuran chromogen ketones are more abundant, including cimifugin and 5-O-methylvisamminoside, among others. Taking the compound cimifugin (Figure S4B) as an example, the cleavage rule of coumarins was introduced below. Cimifugin exhibited [M + H]^+^ at *m*/*z* 307.1177. The parent ion at *m*/*z* 307.1177 lost H_2_O (18 Da) to form the fragment ion at *m*/*z* 289.0778. The parent ion at *m*/*z* 307.1177 further lost H_2_O (18 Da) and 2 CH_3_ (30 Da), resulting in the fragment ion at *m*/*z* 259.0596. The fragment ion at *m*/*z* 221.0412 was generated through the loss of the C_3_H_2_ (38 Da) group from the fragment ion at *m*/*z* 259.0596. The parent ion at *m*/*z* 307.1177 lost C_4_H_8_O (72 Da) to generate the fragment ion at *m*/*z* 235.1186. The fragment ion at *m*/*z* 205.0884 was formed through the loss of CH_2_O (30 Da) from the fragment ion at *m*/*z* 235.1186. Lastly, the fragment ion at *m*/*z* 177.0554 was obtained through the loss of CO (28 Da) from the fragment ion at *m*/*z* 205.0884.

### 2.4. Identification of YPFS Prototype Components In Vivo

**Figure 4:**
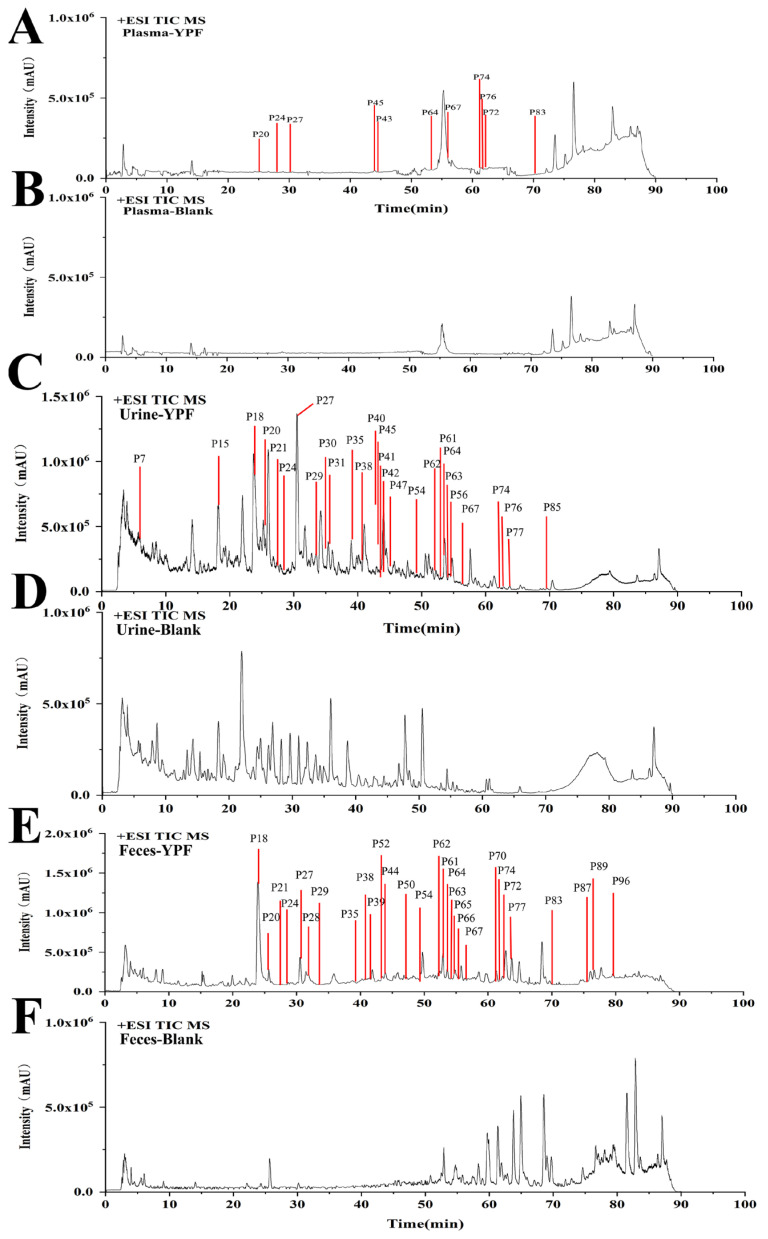
The total ion chromatography (TIC) of YPFS in biological and blank samples in positive ion mode and prototypical components of YPFS in vivo in AR mice. (**A**) YPFS plasma sample; (**B**) blank plasma sample; (**C**) YPFS urine sample; (**D**) blank urine sample; (**E**) YPFS feces sample; (**F**) blank feces sample.

To differentiate prototype components from metabolites, we designated compounds detected in drug-associated biological fluids and YPFS extracts as prototype components. Conversely, compounds exclusively detected in the drug-associated biological fluid but absent in the YPFS extract were classified as metabolites. Under the same analytical conditions, mass spectrometry was utilized to analyze plasma, urine, and feces obtained from AR mice treated with YPFS. To screen for absorbed components, total ion chromatogram (TIC) and extraction ion chromatogram (EIC) curves were employed. Combining the results with those obtained from the in vitro analysis of YPFS, a total of 42 prototype components were identified (Table S5), with 11, 28, and 29 prototype components found in the plasma, urine, and feces, respectively. The base peak chromatograms of YPFS are presented in Figure 4A–F, and the structures of the compounds are shown in Figure S5. These compounds include 12 flavonoids, 11 chromones, 6 saponins, 5 lactones, 3 sesquiterpenoids, 2 coumarins, and 3 other compounds. Figure S6 shows the presence of 42 prototype components across the three biological fluids examined. Six prototype components were commonly detected in all three body fluids: blood, urine, and feces. These components were prim-O-glucosylcimifugin, atractylenolide I, formononetin, astramembrannin II, calycosin-7-O-glucoside, and cimifugin.

### 2.5. Identification of YPFS Metabolites in Vivo

Through the in vitro chemical composition analysis of YPFS, the metabolic pathways of its components were elucidated by cross-referencing data from public databases and literature with the cleavage outcomes for YPFS component precursors. A total of 57 metabolites of YPFS were provisionally identified, including 39 metabolites in plasma, 46 in urine, and 28 in feces. All the identified metabolites are listed in Table S6.

#### 2.5.1. Flavonoid Metabolites

**Figure 5:**
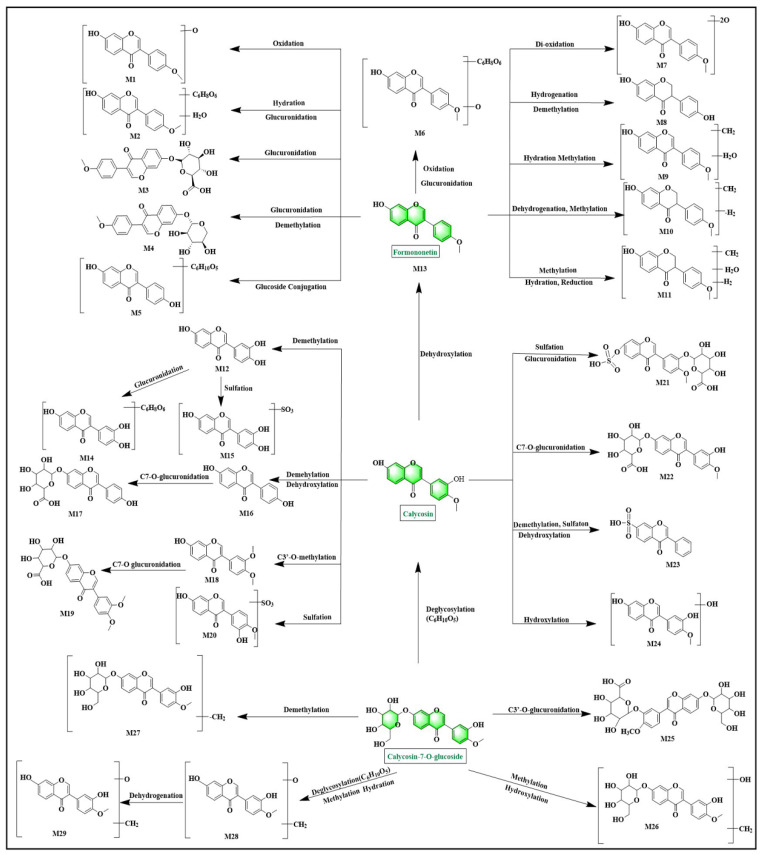
The possible metabolic pathways of formononetin, calycosin, and calycosin-7-O-glucoside from YPFS in AR mice.

As depicted in Figure 5 and Figure S7A, 37 metabolites (M1–M37) were identified as flavonoid-related metabolites in plasma, urine, and feces. These metabolites were primarily associated with formononetin, calycosin, calycosin-7-O-glucoside, ononin, and methylnissolin. The pathway of calycosin’s metabolism was used as an example for clarification.

As depicted in Figure 5, the metabolites of calycosin were identified as M12-M24. Initially, the phase I metabolite-reaction products included M12, M13, M16, and M24. Calycosin exhibited an [M + H]^+^ ion at *m*/*z* 285.0753, and M12 exhibited an [M + H]^+^ ion peak at *m*/*z* 271.0619, which was formed by losing 14 Da reduction compared to calycosin. The fragment ion with *m*/*z* 137.0244 corresponded to the peak of calycosin, suggesting that M12 could be a demethylated derivative of calycosin. M13 displayed an [M + H]^+^ ion peak at *m*/*z* 269.0830, revealing a 16 Da decrease compared to calycosin. Its fragment ion at *m*/*z* 213.0933 aligned with calycosin, leading to the hypothesis that M13 (formononetin) might be a dehydroxylated product of calycosin. M16 presented an [M + H]^+^ ion peak at *m*/*z* 255.0676, demonstrating a 30 Da decrement from calycosin. The fragment ions observed at *m*/*z* 227.0723 and 199.0764 matched those of calycosin, indicating the possibility that M16 was a demethylated and dehydroxylated variant of calycosin. M24 displayed an [M + H]^+^ ion peak at *m*/*z* 301.0734, indicating a 16 Da increase over calycosin. Based on its molecular formula and fragmentation pattern, it was inferred that M24 could be a hydroxylated derivative of calycosin.

Phase II metabolite-reaction products were categorized into methylation, glucuronidation, and sulfation metabolites. M18 showed an [M + H]^+^ ion peak at *m*/*z* 299.0936, indicating a 14 Da increase over calycosin, and the fragment ion of *m*/*z* 197.0655 corresponded to calycosin, suggesting that M18 was a calycosin methylation product.

M14 exhibited an [M + H]^+^ ion peak at *m*/*z* 447.0955, indicating a mass increase of 176 Da over M12. The fragment ions observed at *m*/*z* 225.0702 closely resembled those of calycosin, suggesting that M14 may be the glucuronidation product of M12. Similarly, M17 displayed an [M + H]^+^ ion peak at *m*/*z* 431.1015, demonstrating a mass increase of 176 Da over M16. It was proposed that M17 was the glucuronidation product of M16. M19 showed an [M + H]^+^ ion peak at *m*/*z* 475.123, indicating a mass increase of 176 Da over M18. Combined with fragment ions that were identical to the methylation product of calycosin, M19 was determined to be the glucuronidation product of M18. M22 showed an [M + H]^+^ ion peak at *m*/*z* 461.1082, indicating a mass increase of 176 Da over calycosin. The fragment ions at *m*/*z* 225.0580 and 285.0783 matched those of calycosin, suggesting that M22 could be the glucuronidation product of calycosin.

M15 displayed an [M + H]^+^ ion peak at *m*/*z* 351.018, exhibiting a mass variance of 80 Da from M12. Consequently, it was conceivable that M15 could be a sulfated form of M12. Similarly, M20 displayed an [M + H]^+^ ion peak at *m*/*z* 365.0333, showing a comparable mass increment of 80 Da compared to calycosin. The fragment ions with *m*/*z* values of 253.0498 and 225.0544 coincided with those of calycosin, indicating that M20 might also be a sulfation product of calycosin. M23, with the molecular formula C_15_H_10_O_7_S, showed an [M + H]^+^ ion peak at *m*/*z* 335.0248, displaying a mass increase of 50 Da compared to calycosin. Notably, the fragment ion at *m*/*z* 137.0239 was identical to calycosin, leading to the identification of M23 as a sulfated derivative resulting from the demethylation and dihydroxylation of calycosin. Meanwhile, M21 presented an [M + H]^+^ ion peak at *m*/*z* 541.0647, reflecting a substantial mass augmentation of 256 Da over calycosin. Furthermore, the fragment ion at *m*/*z* 270.0515 aligned with calycosin. Taking into account its molecular formula C_22_H_20_O_14_S, it was hypothesized that M21 could be a sulfated and glucuronidation product of calycosin. These results were consistent with literature indicating that phase II conjugation of calycosin in vivo was very common [22].

#### 2.5.2. Saponins Metabolites

As depicted in Figure S7B, three metabolites (M38-M40) were identified as metabolites of astragaloside IV in plasma, urine, and feces. M38 exhibited an [M + Na]^+^ ion at *m*/*z* 513.3775, indicating a mass 294 Da smaller than astragaloside IV. This observation suggested a molecular formula of C_30_H_50_O_5_ for M38. M38 was determined to be the desugaring product of astragaloside IV, specifically resulting from the removal of glucose (C_6_H_10_O_5_) and xylose (C_5_H_8_O_4_), and was identified as cycloastragenol based on a combination of references and fragmentation patterns. M39 presented an [M + Na]^+^ ion peak at *m*/*z* 511.3277, and its molecular formula C_30_H_48_O_5_ indicated that it was the dehydrogenation product of M38. M40 exhibited an [M + Na]^+^ ion peak at *m*/*z* 591.4888, indicating a mass 80 Da larger than M39 and was inferred to be the sulfation product of M39.

#### 2.5.3. Lactones Metabolites

As depicted in Figure S8A, 13 metabolites (M41–M53) were identified as lactones-related metabolites present in plasma, urine, and feces. These metabolites were primarily associated with atractylenolide I and atractylenolide III. The metabolism pathway of atractylenolide III was used as an illustrative example. M41 exhibited an [M + H]^+^ ion peak at *m*/*z* 265.1433, indicating a mass increase of 16 Da compared to atractylenolide III, suggesting M41 was a hydroxylated derivative of atractylenolide III. M42 displayed an [M + H]^+^ ion peak at *m*/*z* 281.1411, demonstrating a further 16 Da increase from M41, leading to the inference that M42 was a hydroxylated product of M41. M43 presented an [M + H]^+^ ion peak at 263.1291, indicating it was a dehydrogenated derivative of M41. M44 showed an [M + H]^+^ ion peak at *m*/*z* 283.1564, representing a mass increase of 34 Da from atractylenolide III. Combined with its molecular formula C_15_H_22_O_5_, this suggested M44 was a result of hydration and hydroxylation of atractylenolide III. M45 exhibited an [M + H]^+^ ion peak at *m*/*z* 247.1349, demonstrating a 2 Da decrease from Atractonolide III. Its MS/MS ions at 229.1227 and 201.1316 were also 2 Da smaller than atractonolide III, implying M45 was a dehydrogenation product of atractonolide III. M46 displayed an [M + H]^+^ ion peak at *m*/*z* 423.1691, indicating a mass increase of 176 Da from M45, suggesting M46 was a glucuronidation product of M45. M47 showed an [M + H]^+^ ion peak at *m*/*z* 441.1794, demonstrating a further 18 Da increase from M46, inferring that M47 was a hydrated product of M46. These findings were consistent with the literature, which reported hydroxylation and glucuronidation as the primary metabolic reactions of atractonolactone III [22].

#### 2.5.4. Chromone and Coumarin Metabolites

As depicted in Figure S8B, four metabolites (M54–M57) were identified as metabolites of cimifugin and scopoletin in plasma, urine, and feces. M54 exhibited an [M + H]^+^ ion peak at *m*/*z* 323.1151, indicating a mass increase of 16 Da compared to cimifugin. The fragment ions at *m*/*z* 259.0609 and 221.0462 were consistent with cimifugin, suggesting that M54 was a hydroxylated product of cimifugin. M55 displayed an [M + H]^+^ ion peak at *m*/*z* 483.1544, demonstrating a mass increase of 176 Da relative to cimifugin. The fragment ions at *m*/*z* 259.0625 and 235.0621 were consistent with cimifugin, indicating that M55 was the glucuronidation product of cimifugin.

### 2.6. Metabolic Types

As depicted in Figure S9, 39, 46, and 29 metabolites of YPF were detected in plasma, urine, and feces, respectively. In plasma (Figure S10), phase I reactions accounted for 33% (13 metabolites), primarily involving oxidation, reduction, hydroxylation, demethylation, and other reactions. Phase II reactions comprised 41% (16 metabolites), mainly glucuronidation and sulfation reactions. The combined phase I and phase II reactions accounted for 26% (10 metabolites), primarily oxidation–glucuronidation binding and oxidation/reduction/hydration–sulfation/methylation reactions. In urine (Figure S11), phase I reactions accounted for 39% (18 metabolites), mainly demethylation, hydroxylation, oxidation, hydration, and other reactions. Phase II reactions comprised 33% (15 metabolites), primarily glucuronidation and sulfation reactions. The combined phase I and phase II reactions accounted for 28% (13 metabolites), primarily demethylation/oxidation–sulfation/glucuronidation, hydration/reduction–methylation/glucuronidation, and other reactions. In feces (Figure S12), phase I reactions accounted for 50% (14 metabolites), mainly demethylation, hydration, and oxidation. Phase II reactions comprised 21% (6 metabolites), primarily methylation, glucuronidation, and sulfation reactions. The combined phase I and phase II reactions accounted for 29% (8 metabolites), primarily demethylation–methylation/sulfation/glucuronidation reactions.

### 2.7. Network Pharmacology

#### 2.7.1. Construction of YPFS Composition-Targets-AR Networks and Screening of the Key Components

**Figure 6:**
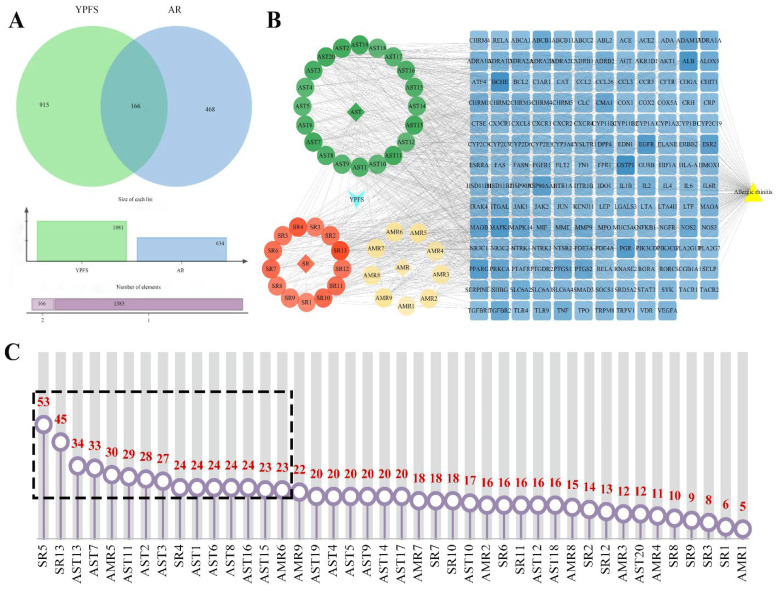
Screening of key components. (**A**) Venn diagram of YPFS and AR miscarriage targets; (**B**) YPFS–active component–target–disease network; (**C**) The degree information from network topology analysis on the 42 compounds.

As depicted in Figure 6A, 1081 YPFS prototype component-related targets and 634 AR-correlated targets were obtained after removing duplicates. Subsequently, 166 overlapping targets between YPFS and AR were identified. To visualize the complex relationships between the active ingredients of YPFS and AR-related targets, a YPFS prototypical component–target–AR network was constructed using Cytoscape v3.9.1. This network comprised 213 nodes (including 42 ingredients, 3 herbs, YPFS, AR, and 166 targets) and 1016 edges (Figure 6B). Table S7 lists the serial numbers of the 42 prototype compounds. In the network, circles represent effective components, blue indicates common targets and yellow represents AR. The 42 components were sorted based on their degree of centrality using Network Analyzer (Figure 6C, Table S8). Ranking of the compounds based on the degree value showed that the top 15 compounds were cimifugin, wogonin, formononetin, skullcapflavone II, atractylenolide III, isoastragaloside I, melilotocarpan B, afrormosin, anomalin, methylnissolin, astramembrannin II, calycosin, apigenin, isomucronulatol, and atractylenolide I. It was predicted that these ingredients may be the key compounds for the treatment of AR with YPFS.

#### 2.7.2. Protein–Protein Interaction (PPI) Analysis and Screening of the Key Targets

**Figure 7:**
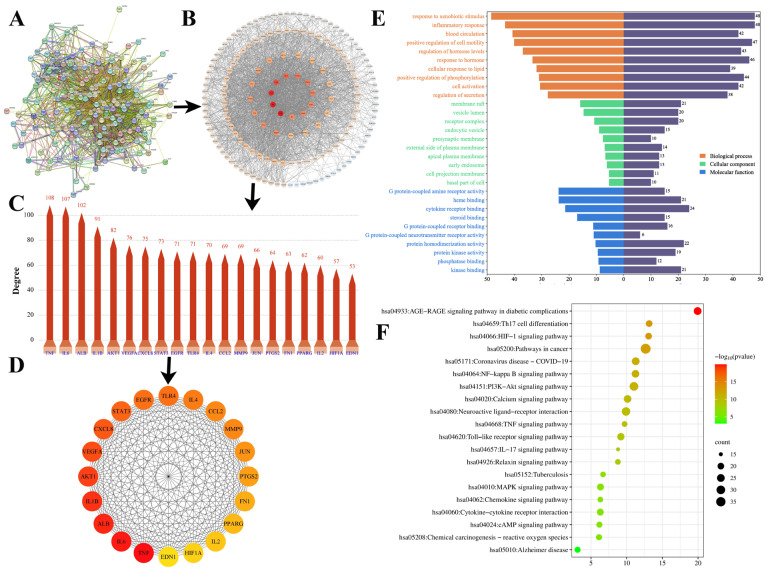
Screening of key targets and KEGG analysis. (**A**) Protein–protein interaction (PPI) network; (**B**) Circle plots for PPI network; (**C**) the degree information of 20 target; (**D**) Circle plots for the 20 targets with the highest degree; (**E**) columnar plots for GO analysis; (**F**) dot bubble plots for KEGG.

The obtained 166 potential genes were imported into String to generate the PPI network, which consisted of 2184 edges (Figure 7A, Figure S13) and was visualized using Cytoscape v3.9.1 (Figure S14). The top 20 genes were identified as key targets using the “Cytohubba” plugin (Figure 7B–D, Table S9). The results showed tumor necrosis factor (TNF), interleukin-6 (IL6), albumin (ALB), interleukin-1β (IL-1β), alpha kinase threonine-1 (AKT1), vascular endothelial growth factor A (VEGFA), c-x-c chemokine ligand 8 (CXCL8), signal transducer and activator of transcription 3 (STAT3), epidermal growth factor receptor (EGFR), toll-like receptor 4 (TLR4), interleukin-4 (IL4), c-c motif chemokine ligand 2 (CCL2), matrix metallopeptidase 9 (MMP9), jun proto-oncogene (JUN), prostaglandin-endoperoxide synthase 2 (PTGS2), fibronectin 1 (FN1), peroxisome proliferator-activated receptor gamma (PPARG), interleukin-2 (IL2), hypoxia-inducible factor 1 subunit alpha (HIF1A) and endothelin 1 (EDN1), suggesting that these targets may be key targets for YPFS in the treatment of AR.

#### 2.7.3. GO and KEGG Analysis

GO analysis identified 1810 biological processes (BP), 98 cell components (CC), and 202 molecular functions (MF). The top 10 enrichments for each of the three GO categories were presented in Figure 7E and Figure S15 and Table S10. BP included inflammatory response, cellular response to lipids, cell activation, and regulation of secretion. CC primarily encompassed the receptor complex, the external side of the plasma membrane, the cell projection membrane, and the basal part of the cell. MF involved cytokine receptor binding, steroid binding, G protein-coupled receptor binding, and kinase binding. KEGG pathway enrichment analysis using DAVID revealed 160 enriched pathways. These genes were particularly enriched in immune and inflammatory-related pathways. The top 20 pathways (Figure 7F and Table S11) encompassed the toll-like receptor signaling pathway, PI3K-Akt signaling pathway, HIF-1 signaling pathway, MAPK signaling pathway, TNF signaling pathway, and NF-kappa B signaling pathway, among others. The greater the number of genes in these pathways, the higher the likelihood that YPFS treated AR by modulating these pathways or biological processes.

### 2.8. Molecular Docking

**Figure 8:**
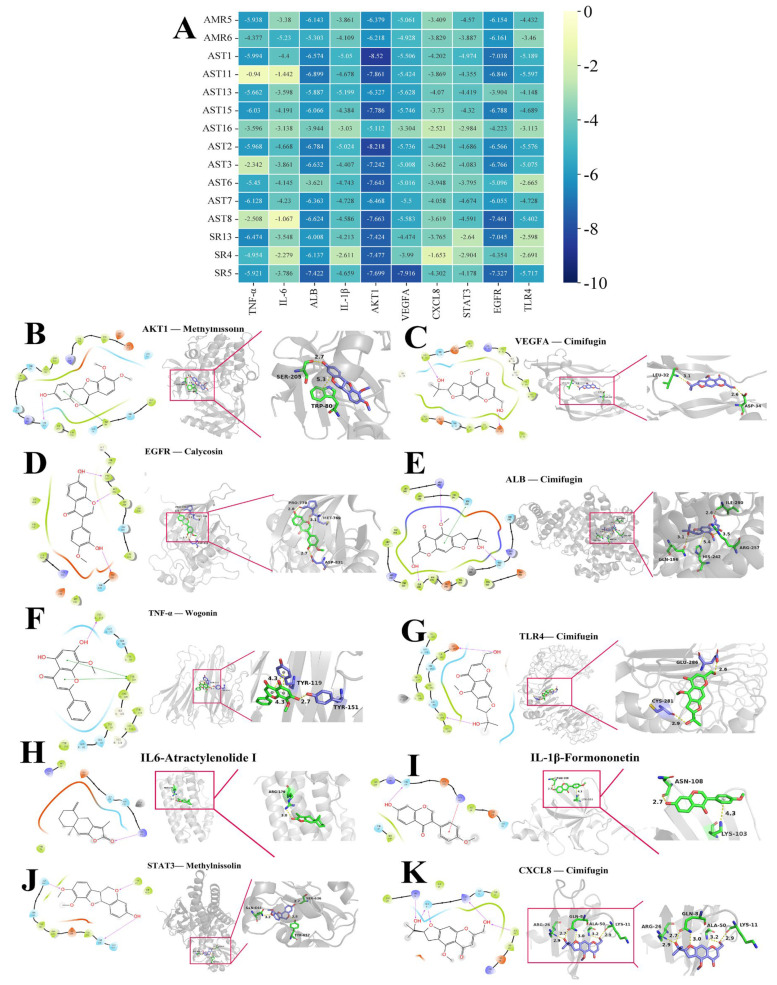
Molecular docking results. (**A**) Heatmap of 15 key components with 10 core targets; (**B**) methylnissolin (AR1)-AKT1; (**C**) cimifugin (SR5)-VEGFA; (**D**) calycosin (AR8)-EGFR; (**E**) cimifugin (SR5)-ALB; (**F**) wogonin (SR13)-TNF-α; (**G**) cimifugin (SR5)-TLR4; (**H**) atractylenolide I (AMR6)-IL6; (**I**) formononetin (AR13)-IL-1β; (**J**) methylnissolin (AR1)-STAT3; (**K**) cimifugin (SR5)-CXCL8.

To elucidate the interactions between compounds and the primary targets of YPFS in the treatment of AR, we conducted molecular docking of the top 10 key targets with 15 core components to investigate the binding modes of protein–ligand complexes. Generally, a higher affinity between receptor and ligand corresponds to a lower score. It was widely accepted that a binding energy less than −5.0 kJ/mol indicates a favorable binding interaction between the compound and target. As depicted in the docking affinity heatmap (Figure 8A, Table S12), the core target proteins AKT1, EGFR, and ALB exhibited binding energies below −5 kJ/mol, indicating strong binding affinities for most of the key components. This implies that they were crucial targets for YPFS in the treatment of AR. Furthermore, Figure 8B–K illustrated that these compounds (methylnissolin, calycosin, cimifugin, atractylenolide I, formononetin, and wogonin) had the highest docking scores at each target, with the specific docking results parameters provided in Table S12 and Table 1. The results revealed that cimifugin (SR5) achieved the lowest binding energy for VEGFA, ALB, TLR4, and CXCL8 among the 15 components (Table S12 and Table 1). Cimifugin formed not only two hydrogen bonds with the amino acid residues ASP:34 and LEU:32 of VEGFA, but also four hydrogen bonds with the amino acid residues ILE:290, ARG:257, HIS:242 and GLN:196 of ALB, two hydrogen bonds with the amino acid residues GLU-286 and CYS-281 of TLR4, and four hydrogen bonds with the amino acid residues ARG:26, GLN:8, ALA:50 and LYS:11 of CXCL8.

Methylnissolin (AST1) showed the highest docking scores for AKT1 and STAT3 among the 15 components (Table S12 and Table 1). This binding stability was closely associated with the efficacy of YPFS in the treatment of AR. Specifically, Methylnissolin exhibited the lowest energy when binding to AKT1, forming a hydrogen bond with the amino acid residue AER-205 of AKT1 and one pi–pi interaction with TRP-80. Atractylenolide I formed one hydrogen bond with amino acid residue ARG:179 of IL6. Calycosin formed three hydrogen bonds with the amino acid residues MET:769, PRO:770, and ASP:831 of EGFR. Formononetin formed two hydrogen bonds with amino acid residues ASN:108 and LYS:103 of IL-1β. One hydrogen bond was formed between wogonin and the amino acid residue TYR:151 of TNF-α, and two π–π bonds were formed with TYR:119. These results suggested that methylnissolin, calycosin, cimifugin, atractylenolide I, formononetin and wogonin may be key substances in improving AR.

### 2.9. Validating the Anti-AR Activity of Active Components (Vitro)

**Figure 9:**
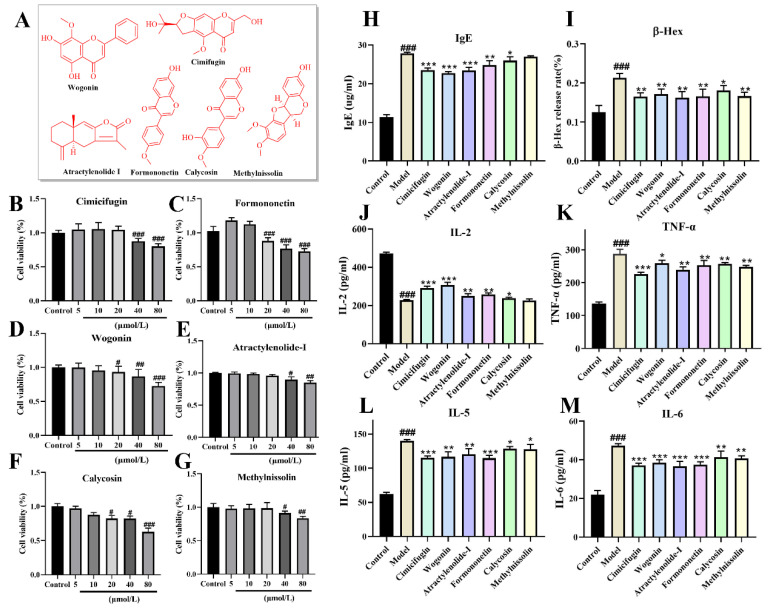
Verification of anti-AR effects of active ingredients on RBL-2H3. (**A**) The structural formulae of six active ingredients; MTT results for cimifugin (**B**), formononetin (**C**), atractylenolide-I (**D**), wogonin (**E**), calycosin (**F**), and methylnissolin (**G**); anti-AR effects of the active ingredients in terms of (**H**) Ig-E, (**I**) Ꞵ-Hex, (**J**) IL-2, (**K**) TNF-ɑ, (**L**) IL-5, and (**M**) IL-6 (^#^ *p* < 0.05, ^##^ *p* < 0.01, **^###^** *p* < 0.001 compared to the control and model group; * *p* < 0.05, ** *p* < 0.01 and *** *p* < 0.001 compared to the model group).

Comprehensive analysis of the vivo composition-based network pharmacological and molecular docking revealed that cimifugin, wogonin, formononetin, methylnissolin, calycosin, and atractylenolide I played key roles, with their structural formulae (red) shown in Figure 9A. The maximum administered doses were maintained at 20 μmol/L for cimifugin, atractylenolide I, and methylnissolin, and the maximum administered doses of formononetin, wogonin, and calycosin were maintained at 10 μmol/L (Figure 9B–G), which did not affect cell viability. As shown in Figure 9H–M, these six potential active ingredients improved the secretion of IgE, β-Hex, and inflammatory factors (IL-2, IL-5, IL-6, and TNF-α) in DNP-IgE/BSA-modeled RBL-2H3 cells to different degrees. Compared with the release rate of IL-6 and TNF-α in the model group, there was a significant reduction in the improvement in the 6 component groups (*p* < 0.01). Compared with the β-Hex, IL-5, IL-2, and IgE release rates in the model group, the rate of improvement was significant in all four component groups except for methylnissolin and calycosin (*p* < 0.01). These results further validated that these compounds may be the key substances for improving AR.

### 2.10. Validating the Anti-AR Activity of Active Components (Vivo)

#### 2.10.1. Active Components Improve Nasal Symptom Score and Serum Indicators

**Figure 10:**
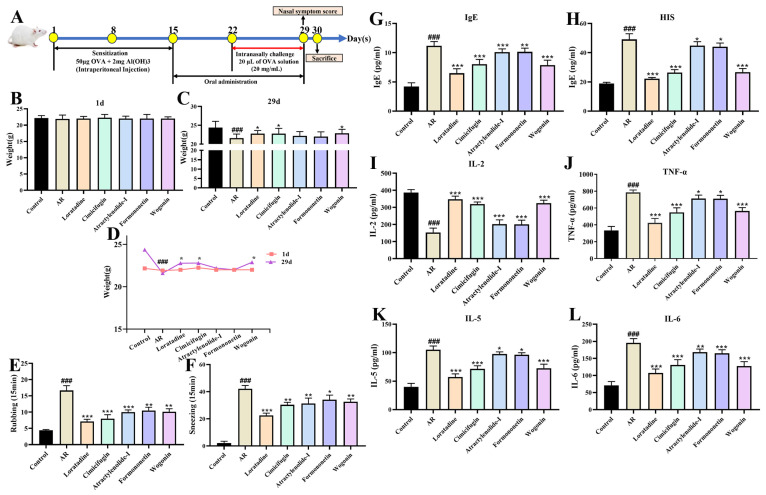
Effects of cimifugin, atractylenolide I, formononetin, and wogonin on body weight, behavior, and serum cytokine levels of AR mice. (**A**) The flow chart of the experiment; (**B**) initial weight; (**C**) last weight; (**D**) weight curve, (**E**) rubbing; (**F**) sneezing, (**G**) IgE; (**H**) histamine; (**I**) IL-2; (**J**) TNF-α; (**K**) IL-5; (**L**) IL-6 (**^###^** *p* < 0.001 compared to the control group; * *p* < 0.05, ** *p* < 0.01 and *** *p* < 0.001 compared to the model group).

After a comprehensive analysis of the cellular validation results, four compounds (cimifugin, wogonin, formononetin, and atractylenolide I) were selected to continue the validation at the animal level (Figure 10). At the conclusion of the 30 d experimental procedure (Figure 10A), the body weight of AR mice was reduced. Compared to the control group, the model group exhibited a significant decrease in body weight (*p* < 0.001). As shown in Figure 10B–D, cimifugin and wogonin significantly enhanced the body weight of AR mice (*p* < 0.05). Compared to the model group, four compounds (cimifugin, wogonin, formononetin, and atractylenolide I) could effectively reduce the number of rubbing and sneezing in AR mice (Figure 10E,F). As shown in Figure 10G–L, compared with the release rate of histamine, IgE, IL-5, IL-6, and TNF-α in the serum of the model group, there was a significant reduction in the improvement of four active components groups (*p* < 0.05). Compared with the release rates of IL-2 in the model group, four compounds (cimifugin, wogonin, formononetin, and atractylenolide I) could significantly increase (*p* < 0.001, Figure 10I). These results further validated that these compounds may be the key substances for improving AR.

#### 2.10.2. Active Components Improve Pathological State of Nasal Mucosa and Lung

**Figure 11:**
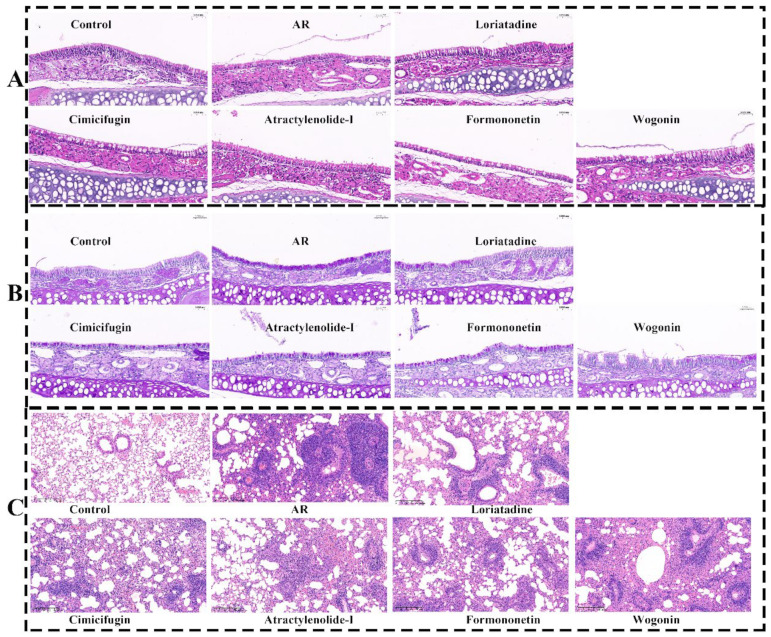
Effect of cimifugin, atractylenolide I, formononetin, and wogonin on histological changes in the nasal mucosa and lung of AR mice. (**A**) HE staining (nasal mucosa, 200×); (**B**) PAS staining (nasal mucosa, 200×); (**C**) HE (lung, 400×).

The staining results of HE and pas indicated that the ciliated structure of the nasal mucosa in the control group was intact, and the epithelial cells were arranged neatly. AR mice were massive cilium shedding in the nasal mucosa, a marked increase in goblet cells, accompanied by inflammatory cell infiltration. Additionally, the epithelial cells showed obvious congestion and edema, with detachment of the basement membrane. Cimifugin, atractylenolide I, formononetin, and wogonin showed significant improvements in nasal mucosa congestion, inflammatory cell infiltration, goblet cell hyperplasia, and cilium shedding (Figure 11A,B).

HE staining revealed normal lung tissue morphology in the control group, with clear alveolar, bronchial, and interstitial structures. In contrast, AR mice showed significant lung tissue enlargement, severe hemorrhage, and extensive inflammatory cell infiltration around the bronchi and alveoli. Cimifugin, atractylenolide I, formononetin, and wogonin exhibited no obvious alveolar hemorrhage or edema, with marked improvement in inflammatory cell infiltration, indicating that active components had a strong improvement effect on the nasal mucosa and lung (Figure 11C).

## 3. Discussion

In this study, we combined the GNPS molecular network with traditional manual identification to systematically identify 98 chemical constituents of YPFS. In total, 42 prototypical components and 57 metabolites were identified in plasma, urine, and feces of AR mice. The relevant metabolic pathways mainly involved reduction, demethylation, hydroxylation, methylation, and glucuronidation. After oral administration, as TCM traverses the digestive tract, numerous metabolic reactions may ensue, triggered by gastric acid, a range of digestive enzymes, and intestinal flora [23]. Many researchers have explored the potential medicinal ingredients of TCM through the dynamic fermentation of human intestinal bacteria [24,25]. The primary metabolic reactions undergone by flavonoids and their glycosides within the body involve hydrolysis, conjugation, cleavage, and oxidation. Our research findings align with these processes. Compared with existing studies on YPFS, this investigation has identified a more comprehensive array of chemical constituents and successfully performed in vivo characterization of both prototype components and their metabolites in AR mice, which substantially contributes to elucidating the holistic material basis underlying YPFS’s therapeutic effects on AR [9]. Furthermore, the established methodology provided a validated framework for systematic compositional analysis and metabolic investigation of other TCM compound formulations.

Subsequently, a YPFS-active component-target-AR network was constructed based on 42 prototype components identified in vivo, and 15 key constituents were prioritized through degree centrality analysis. This was followed by molecular docking between these prioritized components and the top 10 key targets screened from the PPI network. The results indicated that cimifugin, methylnissolin, calycosin, atractylenolide I, formononetin, and wogonin exhibited the lowest energy of binding with each target, suggesting they were potentially the key components of YPFS for treating AR. Concurrently, the results of KEGG and GO analyses suggested that the NF-κB pathway may be a key pathway in the improvement of AR by YPFS. Wang et al. et al. found that YPFS could reduce the levels of NF-κΒ and TSLP in nasal lavage fluid of patients, along with decreased serum concentrations of IL-6 and TNF-α in AR patients through a multicentre clinical trial [26]. This suggested that YPFS may improve AR by modulating the NF-κB/TSLP signaling pathway, in keeping with our study. Through PPI analysis, TLR4, TNF-α, and IL1β were selected among the top 10 core targets with the highest docking scores, and they were also identified as key targets of the NF-κB pathway (Figure S16), indicating their significant role in the treatment of AR by YPFS. TLR4, a pivotal target in the toll-like receptor pathway, has been established in numerous studies as a crucial target in AR [27,28,29]. It played the key role of modulating the NF-κB pathway, resolving the Th1/Th2 imbalance, and suppressing allergic symptoms [30,31]. Hu et al. et al. found that YPFS could suppress the release of inflammatory factors and the apoptosis of cells as well as relieve the symptoms of AR rats probably by inhibiting the TLR4/NF-κB pathway [32]. Nie et al. also explained that YPFS could ameliorate atopic dermatitis by inhibiting the TLR4/MyD88/NF-κB pathway through animal experiments, and molecular docking showed that compounds such as calycosin, formononetin, and divaricatol had specific binding properties with TNF-α, IL-6, and TLR4 [33]. These findings align with our results, which increased the credibility of the key components selected. In a word, the six key potential active components of YPFS for treating AR were screened through network pharmacology and molecular docking.

Conclusively, the pharmacological efficacy of the prioritized compounds was rigorously validated through in vitro bioassays and in vivo functional studies in AR models. Firstly, cellular validation was carried out on six ingredients, and the results showed that compared with the β-Hex, IL-5, IL-2, and IgE release rates in the model group, the rate of improvement was significant in all four component groups except for methylnissolin and calycosin (*p* < 0.01). Then, the efficacy of the four compounds (cimifugin, atractylenolide I, formononetin, and wogonin) was further verified at the animal level, and it was found that could improve nasal symptom score, serum indicators, pathological state of the nasal mucosa and lung of AR mice. Previous studies have demonstrated that cimifugin has the capacity to suppress allergic inflammation by downregulating epithelial-derived factors crucial to the inflammatory process [34]. Research has indicated that wogonin was a potential ingredient active against AR and has identified EGFR and AKT1 as key targets for AR treatment with YPFS [35,36]. Wogonin could reduce the level of IgE, IL-4, and IL-5 in serum, effectively inhibit AR-like symptoms, notably inhibiting the production of TSLP and IL-33, and play an anti-allergic role in AR mice [37]. Cheng et al. also explained that wogonin was the key active component in improving AR integrating analysis of network pharmacology and proteomics [38]. Formononetin could improve IL-13-induced inflammation and mucus formation in human nasal epithelial cells by activating the SIRT1/Nrf2 signaling pathway [39]. Qu et al. also found that formononetin was a potential component for treating allergic rhinitis through network pharmacology and molecular docking [40]. Atractylenolide I, a novel antagonist of TLR4, inhibited TLR4 signaling by disrupting the binding of lipopolysaccharide (LPS) or paclitaxel to TLR4 in human leukocyte membranes [41,42]. This compound subsequently reduced the production of TNF-α, IL-6, and NO and the expression of monocyte chemotactic protein-1 (MCP-1) in vascular smooth muscle cells. Furthermore, atractylenolide I inhibited the activation of p38 mitogen-activated protein kinase (p38-MAPK) and nuclear factor kappa-light-chain-enhancer of activated B cells (NF-κB) [43]. In summary, the vitro (RBL-2H3 cells) and in vivo (AR mice) results further validated that these four compounds (cimifugin, atractylenolide I, formononetin, and wogonin) may be the key substances for improving AR.

## 4. Materials and Methods

### 4.1. Reagents and Instrument

Saline for injection was purchased from Shuanghe Pharmaceutical Co., Ltd. (Huai’an, Jiangsu, China). Ovalbumin, aluminum hydroxide, DNP-BSA (A2831), DNP-IgE (D8406), and 4-nitrophenyl-N-acetyl-β-D-glucosaminide were obtained from Sigma-Aldrich (Saint Louis, MO, USA). The freeze dryer was acquired from Sihuan Scientific Instrument Factory Co. Ltd. (Beijing, China). An electronic analytical balance from Shanghai Huaiping Chemical Co. Ltd. (Shanghai, China) was used. A cryogenic centrifuge from Beckman Coulter (Darmstadt, Germany) was utilized. The laparotomy syringe was procured from Jiangsu Great Wall Medical Co., Ltd. (Nanjing, Jiangsu, China). The Milli-Q ultrapure water system was supplied by Millipore (Bedford, MA, USA). IL-2, IL-5, IL-6, and TNF-α ELISA kits were purchased from Aifang Co., Ltd. (Changsha, Hunan, China). Immunoglobulin E (IgE) and histamine were measured by ELISA using kits from Wuhan Fain Biologicals (Wuhan, China). RBL-2H3 cells were obtained from the Shanghai Chinese Academy of Sciences (Shanghai, China). Dimethyl sulfoxide (DMSO) and MTT were procured from Biyuntian (Shanghai, China). Cimifugin (purity ≥ 98%), formononetin (purity ≥ 98%), atractylenolide-I (purity ≥ 98%), atractylenolide III (purity ≥ 98%), calycosin (purity ≥ 98%), methylnissolin (purity ≥ 98%), and wogonin (purity ≥ 98%) were purchased from Chengdu Efa Biotechnology Co., Ltd. (Chengdu, China).

### 4.2. Extraction of YPFS

*Astragalus mongholicus* Bunge *(AST), Atractylodes macrocephala* Koidz *(AMR)*, and *Saposhni-kovia divaricata* (Turcz.) Schischk *(SR)* were obtained from the Clinic of Nanjing Traditional Chinese Medicine (Nanjing, Jiangsu, China) and authenticated by Prof. Qi Jin of China Pharmaceutical University, who specializes in Traditional Chinese Medicine. AST, AMR, and SR were obtained from Hunyuan (Shanxi, China, 39°41′35.7″ N, 113°41′16.3″ E), Pan’an (Zhejiang, China, 29°3′26.3″ N, 120°26′44.1″ E), Chifeng (Neimenggu, China, 42°15′23.50″ N, 118°52′58.14″ E). AST, AMR, and SR were powdered using a 20-mesh sieve and accurately weighed 180, 90, and 90 g at a ratio of 3:1:1. Then extracted twice with 10 volumes of 95% ethanol for 2 h each. The extract was concentrated using a rotary evaporator and then freeze-dried, resulting in a weight of 89.53 g. The yield was calculated to be 29.84% and stored in a −20 °C freezer for using.

### 4.3. Animal Experiments

Ten female BALB/c mice (18–22 g) were purchased from Yangzhou University (Yangzhou, Jiangsu Province, China) under license number SCXK (Su) 2020-0009. The animals were housed in a room maintained at a temperature of 23 ± 1 °C and subjected to a 12-h light/dark cycle. All the procedures were approved by the Animal Ethics Committee of China Pharmaceutical University.

The AR model was constructed according to the methods described in references [44,45] and consisted of two steps: the basal sensitization step, the ovalbumin (OVA) was used as the sensitizing antigen, and the 200 μL saline containing 50 μg of OVA and 2 mg of aluminum hydroxide with the intraperitoneal injection. Three sensitizations were performed on days 1, 8, and 15 d. The challenge phase began 7 d after the end of the basal sensitization phase and consisted of instilling 20 μL of OVA solution (20 mg/mL) into the bilateral nasal cavity of the mice once a day for 8 consecutive days. The mice were randomly divided into the model group and YPFS group. Saline and YPFS (25 g/kg body weight) were administered to the AR and YPFS groups, respectively, continuously for 3 d from 27 d to 29 d, once in the morning and once in the evening.

### 4.4. Sample Pretreatment

Plasma samples: After the final administration, blood samples were collected from the aforementioned groups at designated time intervals of 0.5, 1, 2, 4, and 8 h, specifically from the posterior venous location behind the eye, in volumes of 500 μL. These samples were transferred into centrifuge tubes containing sodium heparin and centrifuged at 3500 rpm for 10 min. The supernatant plasma was stored in a freezer at −20 °C. Subsequently, the plasma samples were combined with acetonitrile in a 1:3 ratio, vortexed for 1 min, and centrifuged at 4000 rpm for 15 min. The resulting supernatant was evaporated using a nitrogen blower, and the residual material was dissolved in a methanol–water mixture (7:3, *v*/*v*). This solution was then centrifuged at 12,000 rpm for 15 min and filtered through a 0.22 μm microporous filter membrane prior to analysis. The urine and feces sample preparation procedures followed the same protocol as that of the plasma samples.

### 4.5. HPLC and MS Condition

#### 4.5.1. Optimized Detection Conditions

The wavelength (220, 254, and 258 nm), injection volume (2.5, 5, and 10 mg), flow rate (0.6, 0.8, and 1 mL/min), and column temperature (25, 30, and 35 °C) were investigated and optimized.

#### 4.5.2. Conditions of Chromatography and Methodological Study

The Agilent 1260 Infinity HPLC system from Agilent Technologies (Santa Clara, CA, USA) was used for chromatographic separation. A Diamonsil C18 column (4.6 × 250 mm, 5 μm) was employed. The mobile phase consisted of acetonitrile as phase A and water with 0.01% formic acid as phase B. The elution gradient proceeded as follows: 97–85% B for 0–20 min; 85–82% B for 20–25 min; 82–80% B for 25–30 min; 80–72% B for 30–40 min; 72–60% B for 40–50 min; 60–40% B for 50–70 min; 40–5% B for 70–85 min; 5–0% B for 85–90 min. 20 μL sample was injected for analysis.

Precision experiment: An appropriate amount of YPFS sample powder was weighed and prepared for the sample solutions. Under the specified chromatographic conditions, the sample was continuously injected six times. Repeatability experiment: Six test sample solutions were prepared in parallel from the same YPFS sample powder according to the preparation method, followed by HPLC analysis. Stability experiment: HPLC analysis was performed at 0, 2, 4, 8, 12, and 24 h after sample preparation. The relative peak areas of the four compounds (calycosin, atractylenolide III, cimifugin, formononetin) were recorded, and the relative standard deviation (RSD) values were calculated.

#### 4.5.3. Conditions of Mass Spectrometry

Mass spectrometry data were collected using the Agilent 1260 Q-TOF MS/MS system (Agilent Technologies, Santa Clara, CA, USA), which was equipped with an electrospray ionization (ESI) interface. The operational parameters were set as follows: The ESI source was operated in positive mode with a nitrogen (N_2_) flow rate of 10.0 L/min for the drying gas. The temperature of the drying gas was maintained at 350 °C, and the nebulizer pressure was set to 40 psi. The capillary voltage was adjusted to 4000 V, while the skimmer voltage was maintained at 65 V. Additionally, the fragment voltage was set to 135 V, and the MS/MS collision voltage was configured at 30 V. The scan range covered a mass range from 50 to 1500 Da. Furthermore, the detection wavelength was 254 nm. The collected data were subsequently analyzed using the Agilent Mass Hunter Workstation B.06.00 (Agilent Technologies, Santa Clara, CA, USA).

### 4.6. Molecular Networks

The molecular network (MN) of the components of YPFS was constructed following the online process of the GNPS database. The original MS/MS data files were converted into mzML format using the MSConvert software (Institute for Systems Biology, Washington, DC, USA) from the ProteoWizard package, and these mzML format files were subsequently checked using MZmine 2 v.40.1 [46]. The mzML format file was uploaded to the GNPS database using WinSCP software (Prague University of Economics and Business, Prague, Czech Republic,), and the MN of YPFS was established according to the online workflow at GNPS. The parameters for the MN were set with the following specifications: a precursor ion mass error of 0.02 Da, a fragment ion mass error of 0.02 Da, a minimum cosine value of 0.7, a minimum requirement of six paired fragment ions, a maximum limit of ten connections at a single point, a maximum number of compounds in the network of 100, a filter precursor ion window of +/−17 Da, and retention of only the first six peaks in the range of +/−50 Da. The MN was visualized using the online GNPS and Cytoscape 3.9.0 software.

### 4.7. Network Pharmacology

#### 4.7.1. Screening of Prototype Compounds Targets and Disease Targets

TCMSP (ttps://old.tcmsp-e.com/tcmsp.php (accessed on 21 March 2023)), BATMAN-TCM (Score ≥ 10, http://www.badd-cao.net:2345/ (accessed on 21 March 2023)), the Swiss Target Prediction database (http://swisstargetprediction.ch/ (accessed on 21 March 2023)) and PharmMapper (Norm Fit ≥ 0.8, http://www.lilab-ecust.cn/pharmmapper/ (accessed on 21 March 2023)) were used to predict the targets of active ingredients. AR-associated targets were searched for using the keyword “allergic rhinitis” at GeneCards (https://www.genecards.org/ (accessed on 23 March 2023)), TTD (http://db.idrblab.net/ttd/ (accessed on 23 March 2023)) and Drugbank (https://go.drugbank.com/ (accessed on 23 March 2023)). After deduplication, all the targets were combined, and Uniprot (https://www.uniprot.org/ (accessed on 23 March 2023)) was used to convert and correct the ID of the target name for further study. A Venn diagram was used to depict the overlapping genes regarded as potential targets of YPFS therapy for AR.

#### 4.7.2. Compound–Target–Disease Network Construction and Screening of Key Components

The information from the column “network” and “type” from the file in XLXS format was imported to Cytoscape 3.9.1 and the “YPFS-Targets-AR” network. The key ingredients were screened by analyzing the network topology parameters with the “Network Analyzer” tool.

#### 4.7.3. Protein–Protein Interaction (PPI) Network and Screening of Key Targets

The YPF/AR-related targets were imported to String (https://cn.string-db.org/ (accessed on 28 March 2023)), with the species set as “Homo sapiens”; the obtained data were screened with a minimum required interaction score of 0.4. The results were exported in TSV format and imported to Cytoscape3.9.1 to construct the PPI network, and the key target genes were identified using the “Cytohubba” plugin.

#### 4.7.4. GO and KEGG

Gene ontology (GO) annotation analysis involving biological process (BP), cellular component (CC), and molecular function (MF) was carried out using Metascape. David (https://david.ncifcrf.gov/ accessed on 28 March 2023) was used for KEGG pathway enrichment analysis, improving the obtained related genes with the species set as “Homo sapiens”.

### 4.8. Dockings

Molecular docking was performed on key components and their respective targets. The compounds were downloaded in SDF format from PubChem. The 3D structures of AKT1 (PDB ID: 3O96), ALB (PDB ID: 2BX8), CXCL8 (PDB ID: 1ICW), EGFA (PDB ID: 1M17), IL-1β (PDB ID: 6Y8M), IL6 (PDB ID: 1ALU), STAT3 (PDB ID: 6NJS), TLR4 (PDB ID: 2Z63), TNF-α (PDB ID: 2AZ5), and VEGFA (PDB ID: 1MKK) were obtained from the RCSB Protein Data Bank (https://www.rcsb.org/ accessed on 14 April 2023). AutoDock Vina was utilized for molecular docking. Solvent molecules and other non-essential components were removed from the targets using Pymol 2.5 (Schrödinger, LLC, New York, NY, USA), and the structures were visualized.

### 4.9. Validating the Anti-AR Activity of Active Components In Vitro

RBL-2H3 cells were cultured in MEM medium supplemented with 15% FBS at 37 °C in a 5% CO_2_ incubator. The cells were seeded into 96-well plates at a density of 1 × 10^4^ cells/well, and a control group and groups treated with various concentrations of the drug were established. The plates were incubated for 24 h, 100 μL of MEM medium containing different concentrations of the active components was added to each well. Following a 12 h incubation at a constant temperature, the medium was discarded, and 100 μL of MTT solution was added to each well. After 4 h, 150 μL of DMSO was added to each well, and the plates were shaken for 10 min. The OD value at 570 nm was measured with a reference wavelength of 650 nm, and the cell viability was calculated.

RBL-2H3 cells were diluted to 0.5 × 10^4^ cells/well and seeded into 96-well plates by adding 100 μL per well. The plates were incubated for 24 h; the cells were incubated with 0.5 μg/mL DNP-IgE working solution added to each well. Following another 24 h incubation, the cells were rinsed three times with PBS. Six wells were established for each of the following groups: control, model, and drug administration (cimifugin, formononetin, atractylenolide-I, methylnissolin, calycosin, and wogonin). Each well in the control group was supplemented with 100 μL of serum-free MEM medium, while each group received 100 μL of different concentrations of the test solution. After a 12 h incubation, 100 μL of 400 ng/mL DNP-BSA working solution was added to each well, except for the control group, which received an equal volume of basal medium. Then, the plates were incubated for 1 h. The reaction was terminated by incubation at 4 °C for 10 min, and the supernatant from each well was collected for analysis of various indices. RBL-2H3 expresses high-affinity IgE receptors (FcεRI) on the cell surface. When DNP-IgE binds to the receptor, it triggers the aggregation of FcεRI through DNP-BSA cross-linking, activating downstream signaling pathways (e.g., MAPK, Jak-STAT, and TNF pathways), leading to degranulation and inflammatory factor release [47,48]. This process is highly compatible with the pathological features of allergic rhinitis.

### 4.10. Validating the Anti-AR Activity of Active Components In Vivo

Animal models were constructed in the same way as 4.3 Animal experiments. Seventy mice were randomly divided into seven groups: control group, model group (AR), cimifugin group (30 mg/kg), formononetin group (30 mg/kg), atractylenolide-I group (30 mg/kg), wogonin group (30 mg/kg), and loratadine group (1.67 mg/kg). The dose group was administered continuously from 15 d to 29 d, once daily for 14 d. After 2 weeks, mice were executed to collect nasal tissue, serum, and lungs for analysis. The blood was centrifuged at 3500 rpm for 15 min, and the serum was stored in the refrigerator at -80 °C. Body weight was recorded on days 1 d and 29 d. After the last nasal OVA drop, the number of rubbing and sneezing within 15 min was recorded. Morphological and inflammatory infiltration of the nasal mucosa and left lung were observed under the microscope by the HE and PAS staining.

### 4.11. Data Analysis

The chemical compound database for the three herbs in YPFS was established using the TCMSP (https://old.tcmsp-e.com/tcmsp.php accessed on 1 February 2023), PubChem (https://pubchem.ncbi.nlm.nih.gov/ accessed on 1 February 2023), ChemSpider (https://www.chemspider.com/Default.aspx accessed on 1 February 2023) and Web of Science (https://webofscience.clarivate.cn/wos/alldb/basic-search accessed on 1 February 2023) databases, among others, to retrieve compound information for the relevant medicinal materials. The chemical composition of the metabolites was determined through the identification of compounds, pertinent biotransformation information, relevant scholarly works, databases, and the MS/MS spectra of the metabolites. All data were statistically analyzed using GraphPad Prism 8.0.1 software (GraphPad Software Company, San Diego, CA, USA), and experimental data were expressed as mean ± SD and evaluated for variance normality and uniformity. Analysis of variance was used to compare data of each group, the student test was used to compare data of two groups, and the Dunnett test was used to compare data of three groups or more. *p* < 0.05 was considered statistically significant.

## 5. Conclusions

In this study, the chemical constituents of YPFS were firstly comprehensively analyzed using GNPS combined with HPLC–Q-TOF-MS/MS. A total of 98 compounds were identified, and the main cleavage modes of each type of compound were summarized. Next, the active ingredients of YPFS were further identified from the perspective of in vivo processes. A total of 42 prototypical components and 57 metabolites were identified through HPLC–Q-TOF-MS/MS and the metabolites were all derived from the detected prototypical components. The relevant metabolic pathways mainly involved reduction, demethylation, hydroxylation, methylation, and glucuronidation. Based on 42 prototypical components in YPFS, potential ingredients were screened with network pharmacology and molecular docking. A total of 166 potential targets were found. A total of 15 key components were selected based on the topological analysis of TCM–constituent–target network diagrams, and the core targets were screened through the PPI network. Molecular docking of the 15 core components with the first 10 key targets showed that cimifugin, methylnissolin, calycosin, atractylenolide I, formononetin, and wogonin may be the key components of YPFS for the treatment of AR. Finally, cimifugin, atractylenolide I, formononetin, and wogonin were identified as the key compounds for improving AR via the vitro (RBL-2H3 cells) and in vivo (AR mice) validated. In summary, this study provided a reference for exploring the mechanism of YPFS’s anti-AR effects and helped to clarify the overall material basis of YPFS prescription. Furthermore, the dynamics of the four components identified in this study require further in vivo investigation.

## Figures and Tables

**Table 1:** Molecular docking parameters for compounds with first binding energy for 10 core targets.

Compound	Protein-PDBID	Affinity KJ/Mol	Site (xyz)	Bonding
Methylnissolin (AST1)	AKT1-3O96	−8.52	(8.59, −7.1, 13.14)	SER, TRP
Cimifugin (SR5)	VEGFA-1MKK	−7.916	(5, 0, 16)	LEU, ASP
Calycosin (AST8)	EGFR-1M17	−7.461	(21.29, 0.54, 52.36)	MET, PRO, ASP
Cimifugin (SR5)	ALB-2BX8	−7.422	(5.46, −10.15, 7.28)	ILE, ARG, GLN, HIS
Wogonin (SR13)	TNF-α-2AZ5	−6.474	(−19.82, 74.94, 33.46)	TYR
Cimifugin (SR5)	TLR4-2Z63	−5.717	(−0.1, −53.56, 13.23)	GLU, CYS
Atractylenolide I (AMR6)	IL6-1ALU	−5.23	(0, 5, 0)	ARG
Formononetin (AST13)	IL-1β-6Y8M	−5.199	(6.95, 25.46, −9.28)	LYS, ASN
Methylnissolin (AST1)	STAT3-6NJS	−4.974	(13.24, 56.43, 0.24)	SER, GLN, TYR
Cimifugin (SR5)	CXCL8-1ICW	−4.302	(−6, 29, 30)	ARG, GLN, ALA, LYS

## Data Availability

All data are contained in the article and the Supplementary Materials.

## Supplementary Materials

The following supporting information can be downloaded at: https://www.mdpi.com/article/10.3390/ph18040540/s1, Table S1. Precision investigation results of 4 components of YPFS. Table S2. Repeatability investigation results of 4 components of YPFS. Table S3. Stability investigation results of 4 components of YPFS. Table S4. Mass spectrometric information of compounds from YPFS by HPLC-Q-TOF-MS/MS. Table S5. Mass spectrometric information of prototype components in vivo of YPFS by HPLC-Q-TOF-MS/MS. Table S6. Mass spectrometric information of metabolites components in vivo of YPFS by HPLC-Q-TOF-MS/MS. Table S7. The serial number of the 42 prototype compounds. Table S8. The information from network topology analysis on the 42 compounds. Table S9. The degree information from PPI on the top 20 core targets. Table S10. The detailed information on the top 30 GO terms. Table S11. The details on the top 20 KEGG pathways. Table S12. Molecular docking binding energy (KJ/mol). Figure S1. Optimization of chromatographic conditions. (A. Wavelength; B. Injection volume; C. Flow rate; D. Column temperature). Figure S2. Molecular network of the constituents in YPFS. The node color indicated the chemical type of the compound (red: flavonoids; green: saponins; purple: coumarin; blue: lactone; pink: amino acids; orange: others; pale yellow: organic acids). Figure S3. Chemical fragmentation pathways of chemical components in YPFS. (A) ononin; (B) psoralen. Figure S4. Chemical fragmentation pathways of chemical components in YPFS. (A) imperatorin; (B) cimifugin. Figure S5. The structural formula of prototypical components from YPFS in AR mice. Figure S6. Venn diagram detailing the proportions of plasma, urine and feces. Figure S7. The possible metabolic pathways of (A) ononin and methylnissolin, and (B) astragaloside IV from YPFS in AR mice. Figure S8. The possible metabolic pathways of (A) atractylenolide I and atractylenolideIII, and (B) cimfugin and scopoletin from YPFS in AR mice. Figure S9. The proportion of metabolic types of YPFS in the plasma, urine, and feces of AR mice. Figure S10. Metabolic types of YPFS in the plasma of AR mice. Figure S11. Metabolic types of YPFS in the urine of AR mice. Figure S12. Metabolic types of YPFS in the feces of AR mice. Figure S13. Protein-protein interaction (PPI) network. Figure S14. Circle plots for PPI network. Figure S15. Circle plots for GO analysis. Figure S16. NF-κB pathway diagram with genes predicted to be YPF-AR targets (red).
