# Supplementary material for: Identification of Active Markers of Chinese Formula Yupingfeng San by Network Pharmacology and HPLC-Q-TOF–MS/MS Analysis in Experimental Allergic Rhinitis Models of Mice and Isolated Basophilic Leukemia Cell Line RBL-2H3
Source: Pharmaceuticals (Basel). 2025 Apr 7;18(4):540. doi: 10.3390/ph18040540 (PMC12030416; doi:10.3390/ph18040540)
Supplement: Supplementary file 1 [file pharmaceuticals-18-00540-s001.zip › pharmaceuticals-3513118-supplementary.pdf]

Supplementary Materials

Table S1. Precision investigation results of 4 components of YPFS.

Table S2. Repeatability investigation results of 4 components of YPFS.

Table S3. Stability investigation results of 4 components of YPFS.

Table S4. Mass spectrometric information of compounds from YPFS by HPLC-Q-TOF-MS/MS.

Table S5. Mass spectrometric information of prototype components in *vivo* of YPFS by HPLC-Q-TOF-MS/MS.

Table S6. Mass spectrometric information of metabolites components in *vivo* of YPFS by HPLC-Q-TOF-MS/MS.

Table S7. The serial number of the 42 prototype compounds.

Table S8. The information from network topology analysis on the 42 compounds.

Table S9. The degree information from PPI on the top 20 core targets.

Table S10. The detailed information on the top 30 GO terms.

Table S11. The details on the top 20 KEGG pathways.

Table S12. Molecular docking binding energy (KJ/mol).

Figure S1. Optimization of chromatographic conditions. (A. Wavelength; B. Injection volume; C. Flow rate; D. Column temperature).

Figure S2. Molecular network of the constituents in YPFS. The node color indicated the chemical type of the compound (red: flavonoids; green: saponins; purple: coumarin; blue: lactone; pink: amino acids; orange: others; pale yellow: organic acids).

Figure S3. Chemical fragmentation pathways of chemical components in YPFS. (A) ononin; (B) psoralen.

Figure S4. Chemical fragmentation pathways of chemical components in YPFS. (A) imperatorin; (B) cimifugin.

Figure S5. The structural formula of prototypical components from YPFS in AR mice.

Figure S6. Venn diagram detailing the proportions of plasma, urine and feces.

Figure S7. The possible metabolic pathways of (A) ononin and methylnissolin (B) astragaloside IV from YPFS in AR mice.

Figure S8. The possible metabolic pathways of (A) atractylenolide I and atractylenolideIII (B) cimfugin;and scopoletin from YPFS in AR mice.

Figure S9. The proportion of metabolic types of YPFS in the plasma, urine, and feces of AR mice.

Figure S10. Metabolic types of YPFS in the plasma of AR mice.

Figure S11. Metabolic types of YPFS in the urine of AR mice.

Figure S12. Metabolic types of YPFS in the feces of AR mice.

Figure S13. Protein-protein interaction (PPI) network.

Figure S14. Circle plots for PPI network.

Figure S15. Circle plots for GO analysis.

Figure S16. NF-κB pathway diagram with genes predicted to be YPF-AR targets (red).

Table S1. Precision investigation results of 4 components of YPFS

	Calycosin	Atractylenolide III	Cimifugin	Formononetin
1	28406.300	1362.300	31413.100	19197.600
2	28399.200	1360.100	31399.800	19259.700
3	28403.900	1380.800	31455.000	19310.300
4	28560.000	1322.300	31528.500	19823.800
5	28641.900	1301.000	31521.500	19961.000
6	28910.700	1334.100	31505.800	19026.500
Mean	28553.667	1343.433	31470.617	19429.817
RSD (%)	0.706	2.199	0.178	1.922

Table S2. Repeatability investigation results of 6 components of YPFS

	Calycosin	Atractylenolide III	Cimifugin	Formononetin
1	27931.400	1337.000	31076.600	17958.200
2	27728.300	1333.000	30909.700	17924.400
3	27808.100	1342.000	30934.200	18037.200
4	27717.000	1329.700	30828.300	17025.300
5	28080.800	1318.600	30955.600	17516.200
6	27621.100	1324.400	30855.700	18534.000
Mean	27814.450	1330.783	30926.683	17832.550
RSD (%)	0.599	0.637	0.283	2.870

Table S3. Stability investigation results of 6 components of YPFS

	Calycosin	Atractylenolide III	Cimifugin	Formononetin
1	27856.100	1396.700	31134.000	16682.800
2	27912.300	1393.600	31077.200	16734.700
3	27930.100	1477.400	31192.300	16762.700
4	28499.100	1432.400	31175.000	16911.800
5	28473.100	1425.400	31185.700	16963.700
6	28565.700	1418.900	31169.800	16992.400
Mean	28206.067	1424.067	31155.667	16841.350
RSD (%)	1.199	2.134	0.139	0.776

Table S4. **Mass spectrometric information of compounds from YPFS by HPLC-Q-TOF-MS/MS.**

No.	t_R (min)	[M+H] $^+$/[M+Na] $^+$	Identification	Formula	Mass Error (ppm)	Fragment ions	Source	Classification
P1	2.658	177.0975	Structural analog of arginine	$C_5H_{12}N_4O_3$	4.07	160.0679、 118.0493、 102.0547	AST	Amino acids
P2	2.859	175.1176	Arginine	$C_6H_{14}N_4O_2$	7.77	130.0965、116.0689、70.0649	AST	Amino acids
P3	2.993	116.0708	Proline	$C_5H_9NO_2$	-1.69	70.0659	AST	Amino acids
P4	4.937	365.1061(+Na）	Sucrose	$C_{12}H_{22}O_{11}$	-1.95	365.1060、203.0530	AST	Others
P5	5.272	182.0815	Tyrosine	$C_9H_{11}NO3$	-1.82	165.0566、136.0762、123.0446	AMR	Amino acids
P6	5.54	132.1018	L-Isoleucine	$C_6H_{13}NO_2$	2.33	69.0701、86.8477	AST	Amino acids
P7	5.607	268.1033	Adenosine	$C_{10}H_{13}N_5O_4$	1.61	136.0614	AST	Others
P8	9.226	120.0805	2-ethenylbenzenamine	C_8H_9N	2.32	103.0544、91.0542、77.0379	AMR	Others
P9	9.427	166.0859	Phenylalanine	$C_9H_{11}NO_2$	2.15	120.0818、103.0544	AST	Amino acids
**P10**	**9.469**	**156.0768**	**Histidine**	$C_6H_9N_3O_2$	**-0.3**	**83.0296、111.0657、110.0792**	**AST**	**Amino acids**
P11	11.103	384.1157	Succinoadenosine	$C_{14}H_{17}N_5O_8$	-1.86	252.0718	AST	Others
P12	13.801	367.1513	1-β-D-Glucopyranosyl-L-tryptophan	$C_{17}H_{22}N_2O_7$	-3.61	349.1400、 332.1157、 229.0981、 188.0699、 156.0757、 146.0709	AST	Amino acids
P13	14.387	205.0969	Tryptophan	$C_{11}H_{12}N_2O_2$	1.25	188.0702	AST	Amino acids
P14	14.605	133.0612	Asparagine	$C_4H_8N_2O_3$	3.2	116.0378, 87.0563	**AST**	**Amino acids**
P15	14.815	355.095	Undulatoside A	$C_{16}H_{18}O_9$	-9.72	193.0496、153.0905	SR	Others
**P16**	**17.356**	**355.1024**	**Chlorogenic acid***	$C_{16}H_{18}O_9$	**-0.12**	**163.0386、145.0241、135.038**	**AST**	**Others**
**P17**	**24**	**369.118**	**4-O-feruloyl-D-quinic acid**	$C_{17}H_{20}O_9$	**0.02**	**177.0542、145.0275、117.0328、149.0618、89.0395**	**AST**	**Organic acids**

P18	24.429	321.0978	Divaricatacid	$C_{16}H_{16}O_7$	-2.88	273.0418	SR	Chromone
P19	25.267	193.0845	4-methoxy-6-prop-2-enyl-1,3-benzodioxole	$C_{11}H_{12}O_3$	7.4	118.0431、115.0522、105.0301、103.0560	AST	Others
P20	25.929	469.1697	Prim-O-glucosylcimifugin	$C_{22}H_{28}O_{11}$	1.58	307.1172、290.1118、235.0572	SR	Chromone
P21	27.195	193.0491	Scopoletin	$C_{10}H_8O_4$	2.27	178.0252、161.0227、122.0774、133.1014、137.0632、149.0279、150.076、165.0816、105.0329	SR	Coumarin
P22	28.218	273.076	Naringenin	$C_{15}H_{12}O_5$	0.9	189.0539, 153.0183, 147.0457	AST	Flavonoids
**P23**	**28.251**	**447.129**	**Glycitin***	$\mathbf{C_{22}H_{22}O_{10}}$	**-3.2**	**285.0755、 270.0524、 253.0499、 225.0549、 213.0537、 137.0224**	**AST**	**Flavonoids**
P24	28.486	447.1283	Calycosin-7-O-glucoside	$C_{22}H_{22}O_{10}$	-1.3	285.0754, 270.0522, 253.0496, 137.026	AST	Flavonoids
**P25**	**30.051**	**453.176**	**5-O-methylvisamminoside**	$\mathbf{C_{22}H_{28}O_{10}}$	**1.27**	**291.1230、273.11、219.07、243.07**	**SR**	**Chromone**
**P26**	**30.765**	**219.133**	**Abrine**	$\mathbf{C_{12}H_{14}N_2O_2}$	**-0.9**	**132.1006**	**AST**	**Others**
P27	31.167	307.1177	Cimifugin*	$C_{16}H_{18}O_6$	-0.28	259.0596、221.0412、177.0554、205.0884、235.1186、289.0778	SR	Chromone
P28	32.828	217.1545	Atractylone	$C_{15}H_{20}O$	5.51	159.1158	AMR	Sesquiterpene
P29	34.177	333.1335	3-O-propiony lhamaudol	$C_{18}H_{20}O_6$	0.9	277.5123、205.2236	SR	Chromone
P30	35.25	463.1242	Pratensein-7-O-glucoside	$C_{22}H_{22}O_{11}$	-1.54	301.0703	AST	Flavonoids
P31	35.736	533.129	Calycosin-7-O-Glc-6"-O-malonate	$C_{25}H_{24}O_{13}$	-0.06	285.0743	AST	Flavonoids
P32	39.582	489.1383	Calycosin-7-O-Glc-6"-O-acetate	$C_{24}H_{24}O_{11}$	1.72	285.0746	AST	Flavonoids
P33	39.984	223.0626	Fraxidin	$C_{11}H_{10}O_5$	-2.91	208.2114	SR	Coumarin
**P34**	**40.336**	**247.08**	**Marmesin***	$\mathbf{C_{14}H_{14}O_4}$	**1.97**	**247.0956、175.0383、229.0851、176.0441、147.0466**	**SR**	**Coumarin**
P35	40.453	431.134	Ononin	$C_{22}H_{22}O_9$	-0.79	137.0981、213.133、237.0484、254.0578、269.0797	AST	Flavonoids

**P36**	**40.600**	**247.096**	**2-(2-hydroxypropan-2-yl)-2,3-dihydrofuro[3,2-g] chromen-7-one**	**$C_{14}H_{14}O_4$**	**4.08**	**229.0850、175.0382、91.0540**	**AST**	**Others**
**P37**	**40.956**	**302.3054**	**Tetradecyldiethanolamine**	**$C_{18}H_{39}NO_2$**	**-0.15**	**284.0605、106.0395**	**AST**	**Others**
P38	40.99	293.1018	(3S)-8-(Hydroxymethyl)-3,5-dihydroxy-2,2-dimethyl-3,4-dihydro-2H,6H-benzo[1,2-b:5,4-b'] dipyran-6-one	$C_{15}H_{16}O_6$	0.56	257.0781、275.0912	SR	Chromone
P39	41.325	291.1227	5-O- methylvisamminol	$C_{16}H_{18}O_5$	-0.34	273.1048、233.086、205.0503	SR	Chromone
P40	43.120	271.0599	Apigenin	$C_{15}H_{10}O_5$	-0.7	253.0516, 243.0666, 229.0882,	AST	Flavonoids
P41	43.857	285.0757	Wogonin*	$C_{16}H_{12}O_5$	0.18	271.0538、270.0517	SR	Flavonoids
P42	44.125	439.1613	Sec-O-glucosylhamaudol	$C_{21}H_{26}O_{10}$	-3.26	277.107、259.0971、241.0859、231.0921、217.0505	SR	Chromone
P43	44.528	439.1599	Cnidimoside A*	$C_{21}H_{26}O_{10}$	-0.06	205.0504、259.0968、277.1065	SR	Coumarin
P44	44.528	285.0753	Calycosin*	$C_{16}H_{12}O_5$	1.58	137.0221、197.0717、213.0528、225.1409、253.0475	AST	Flavonoids
P45	44.662	303.1221	Isomucronulatol	$C_{17}H_{18}O_5$	1.99	167.0681、123.0444	AST	Flavonoids
P46	45.064	315.0872	Dihydroxy-dimethoxyisoflavone	$C_{17}H_{14}O_6$	-2.82	300.0648、283.0556、255.0426、167.0634	AST	Flavonoids
P47	45.458	517.1342	Formononetin-7-O-Glc-6"-O-malonate	$C_{25}H_{24}O_{12}$	-0.29	254.0561、137.0528	AST	Amino acids
P48	47.026	295.0394	Isoparsleyin B	$C_{18}H_{14}O_4$	3.49	295.1542、241.1512	SR	Coumarin
P49	47.294	187.0392	Psoralen*	$C_{11}H_6O_3$	-1.23	115.0599、131.0495、143.045、159.1161	SR	Coumarin
P50	47.562	947.5213	Astragaloside VI	$C_{47}H_{78}O_{19}$	-0.31	455.3489、437.3395	AST	Saponins
**P51**	**47.720**	**447.129**	**Sissotrin**	**$C_{22}H_{22}O_{10}$**	**0**	**286.0780、285.0760、123.0440、270.0520**	**AST**	**Flavonoids**
P52	47.763	287.0918	Melilotocarpan B	$C_{16}H_{14}O_5$	-1.4	255.0655、177.0543、153.0544、138.0297	AST	Flavonoids
P53	48.902	473.1439	Formononetin-7-O-Glc-6"-O-acetate	$C_{24}H_{24}O_{10}$	0.69	269.0802、254.0515、	AST	Flavonoids

P54	49.640	389.2553	Norcaperatic acid	$C_{20}H_{36}O_7$	-4.95	371.2444、330.2112、311.2117、284.2021、244.1711	AMR	Amino acids
P55	50.511	301.0723	Pratensein	$C_{16}H_{12}O_6$	-5.45	286.0478、269.0459、241.0472、153.017	AST	Flavonoids
P56	51.141	301.1057	Methylnissolin	$C_{17}H_{16}O_5$	4.5	241.1546、191.0698、167.0698、152.0447、123.0423	AST	Flavonoids
P57	51.409	207.0641	Scoparone	$C_{11}H_{10}O_4$	5.27	191.1272、163.0777、151.0788、107.0786	AMR	Lactone
P58	52.591	217.0491	Xanthotoxin	$C_{12}H_8O_4$	2.01	202.0258、174.0299	SR	Coumarin
P59	52.859	231.1012	Ostenol	$C_{14}H_{14}O_3$	1.56	189.1254、175.4413	SR	Coumarin
P60	52.859	247.0605	Isopimpinellin*	$C_{13}H_{10}O_5$	-1.63	232.037、217.0312	SR	Coumarin
P61	52.926	277.1064	Hamaudol	$C_{15}H_{16}O_5$	2.35	259.0961、244.0705、205.0481、189.0572、177.0506	SR	Chromone
P62	53.328	807.4484 (+Na）	Astragaloside IV	$C_{41}H_{68}O_{14}$	2.2	455.3528、437.3204、419.3309、438.3428	AST	Saponins
P63	54.199	299.0919	Afrormosin	$C_{17}H_{14}O_5$	-1.68	283.0628、137.0560	AST	Flavonoids
P64	54.468	269.0813	Formononetin*	$C_{16}H_{12}O_4$	-1.73	253.0482、254.0751、237.0542、225.5773、213.0913	AST	Flavonoids
P65	54.669	375.1085	Skullcapflavone II	$C_{19}H_{18}O_8$	2.8	360.0845, 345.0618, 327.0517	AST	Flavonoids
P66	55.473	849.4612 (+Na)	Isoastragaloside II*	$C_{43}H_{70}O_{15}$	0.6	789.4391	AST	Saponins
P67	55.942	645.3992(+Na）	Astramembrannin II	$C_{35}H_{58}O_9$	-3.05	437.3216	AST	Saponins
P68	57.149	943.5265	Soyasaponin I	$C_{48}H_{78}O_{18}$	-0.43	797.4654、599.3922、441.3748、423.3597	AST	Saponins
P69	60.814	849.4601 (+Na）	Astragaloside II	$C_{43}H_{70}O_{15}$	0.72	453.3367、157.0519	AST	Saponins
P70	61.082	891.4706 (+Na)	Isoastragaloside I*	$C_{45}H_{72}O_{16}$	0.76	831.454	AST	Saponins
P71	61.35	891.4716 (+Na）	Astragaloside I	$C_{45}H_{72}O_{16}$	-0.39	591.3734、380.1688	AST	Saponins
P72	62.155	249.148	Atractylenolide III*	$C_{15}H_{20}O_3$	2.1	163.0746、175.0745、185.1294、189.0927、203.1437、213.1277、231.1374	AMR	Lactone
P73	62.356	231.1015	2,3-Dihydro-2,2,3-trimethyl-4H-4-one	$C_{14}H_{14}O_3$	-1.2	175.0383、147.0453	AMR	Lactone

P74	62.423	231.1379	Atractylenolide I*	$C_{15}H_{18}O_2$	0.24	105.0706、133.101、159.1173、161.0603、189.0917、203.1432、213.1323	AMR	Lactone
**P75**	**62.515**	**249.1485**	**Reynosin***	$C_{15}H_{20}O_3$	**0.08**	**105.0699、91.0539、119.0854、131.0853、145.0991**	**AMR**	**Lactone**
P76	62.892	249.1483	6-hydroxy atractylenolide I	$C_{15}H_{20}O_3$	0.89	231.1386、213.1282、203.1446	AMR	Lactone
P77	63.696	230.1547	atractylenolactam	$C_{15}H_{19}NO$	-3.31	214.1236、200.1069、174.092	AMR	Lactone
**P78**	**64.233**	**467.194 (+Na）**	**Aurantiamide acetate**	$C_{27}H_{28}N_2O_4$	**0.41**	**91.5057、94.1165**	**AST**	Others
P79	65.206	319.1172	3-O-acetylhygrophenol	$C_{17}H_{18}O_6$	1.3	217.0493	SR	Others
P80	65.809	233.0444	5-hydroxy-8-methoxypsoralen	$C_{12}H_8O_5$	1.17	218.456、162.4132	SR	Coumarin
P81	65.876	271.0956	Imperatorin	$C_{16}H_{14}O_4$	3.28	175.0361、91.5049、119.0856、129.0552、131.1069、147.0458、157.0509、159.0965、185.09、203.033	SR	Coumarin
P82	68.006	329.1383	Proanthophyllin	$C_{19}H_{20}O_5$	2.3	247.5232、214.7864	SR	Coumarin
P83	70.027	233.1531	Atractylenolide II*	$C_{15}H_{20}O_2$	1.75	133.0638、151.0747、159.0793、177.0906、187.1481、215.1433	AMR	Lactone
P84	70.295	233.1484	Thr-Leu	$C_{10}H_{20}N_2O_4$	5.1	187.1432、132.0848、56.0534	AST	Others
P85	70.965	233.1543	3β-hydroxyatractylone	$C_{15}H_{20}O_2$	-2.99	171.0895、159.0819、145.1014	AMR	Sesquiterpene
**P86**	**74.584**	**315.086**	**9s,13r-12-Oxophytodienoic Acid**	$C_{18}H_{28}O_3$	**0.23**	**316.2009、315.1927**	**AST**	**Organic acids**
P87	75.322	203.1802	Aromadendrene, dehydro-	$C_{15}H_{22}$	-3.82	105.0702	AMR	Sesquiterpene
**P88**	**75.472**	**279.2327**	**Linolenic acid**	$C_{18}H_{30}O_2$	**-3.03**	**67.0551、81.0448、93.0859、123.0808、149.0954**	**AST**	**Organic acids**
P89	76.126	253.1196	Ser-Phe	$C_{12}H_{16}N_2O_4$	-5.22	207.1177	AST	Others
**P90**	**76.218**	**231.338**	**Dehydrocostus lactone**	$C_{15}H_{18}O_2$	**-0.19**	**185.1326、143.0847、143.085、231.1376、145.101**	**AMR**	**Lactone**
P91	78.405	219.1753	Sesquiterpenoids	$C_{15}H_{22}O$	-4.39	201.1623、163.1115、109.0993、81.0709	AMR	Sesquiterpene
P92	78.472	339.1981	Canrenone	$C_{22}H_{28}O_3$	4.5	183.0101	AST	Others

P93	78.673	217.18	8β-Ethoxyatractylenolide	$C_{17}H_{24}O3$	-0.65	231.1375、175.0746、163.0744、143.0873	AMR	Lactone
P94	78.74	359.1489	3-O-angeloyl hamaudol	$C_{20}H_{22}O_6$	0.04	259.0961、217.0492、189.0543	SR	Chromone
P95	79.343	279.16	Structural analog of Atractylenolide II	$C_{16}H_{22}O_4$	-3.29	149.023、121.0284	AMR	Lactone
P96	80.148	449.1594 (+Na）	Anomalin	$C_{24}H_{26}O_7$	-5.46	349.1048、327.1244	SR	Coumarin
**P97**	**83.247**	**338.3417**	**Erucamide**	$\mathbf{C_{22}H_{43}NO}$	**0.12**	**321.3142、303.305、339.3454、338.3411、149.1329**	**AST**	**Others**
P98	86.314	256.2635	Palmitamide	$C_{16}H_{33}NO$	-0.03	102.0917、88.0762	AST	Others

Note: Subscript with a horizontal line indicates that those were identified by both manual and molecular networks, and bolded font indicates that those were predicted by molecular networks.

* confirmed by authentic standards.

Table S5. **Mass spectrometric information of prototype components in vivo of YPFS by HPLC-Q-TOF-MS/MS.**

No.	t_R (min)	exp	pre	Identification	Formula	**Mass Error (ppm)**	Fragment ions	Source	Classification	Plasma	Urine	Feces
P7	5.81	268.1057	268.104	Adenosine	$C_{10}H_{13}N_5O_4$	-6.25	136.0614	AST	Saponins		+	
P15	18.075	355.1058	355.1024	Undulatoside A	$C_{16}H_{18}O_9$	-9.72	193.0496、153.0905	SR	Chromone		+	
P18	23.538	321.0998	321.0969	Divaricatacid	$C_{16}H_{16}O_7$	-9.12	273.0959、263.0576、59.0515、	SR	Chromone		+	+
P20	25.59	469.1745	469.1704	Prim-O-glucosylcimifugin	$C_{22}H_{28}O_{11}$	-8.68	307.1172、290.1118、235.0572	SR	Chromone	+	+	+
P21	27.307	193.0513	193.0495	Scopoletin	$C_{10}H_8O_4$	-9.19	178.0274、137.0592、133.0289、79.0560	SR	Coumarin		+	+
P24	29.46	447.1321	447.1286	Calycosin-7-O-glucoside	$C_{22}H_{22}O_{10}$	-7.91	285.0777、195.0794	AST	Flavonoids	+	+	+
P25	33.566	453.1795	453.1755	5-O-methylvisamminoside	$C_{22}H_{28}O_{10}$	-8.79	291.1243、273.1156、145.0679	SR	Chromone		+	+
P27	30.952	307.1197	307.1176	Cimifugin	$C_{16}H_{18}O_6$	-6.81	259.0632、235.0614、79.0551	SR	Chromone	+	+	+
P28	31.824	217.1578	217.1587	Atractylone	$C_{15}H_{20}O$	4.13	159.1158	AMR	Sesquiterpene			+
P30	35.033	463.1278	463.1235	Pratensein-7-O-glucoside	$C_{22}H_{22}O_{11}$	-9.33	301.0703	AST	Flavonoids		+	
P31	35.569	533.1271	533.129	Calycosin-7-O-Glc-6"-O-malonate	$C_{25}H_{24}O_{13}$	3.51	285.0743	AST	Flavonoids		+	

P35	40.47	431.1378	431.1337	Ononin	$C_{22}H_{22}O_9$	-9.63	269.0830、56.0506	AST	Flavonoids		+	+
P38	40.939	293.1045	293.102	(3S)-8-(Hydroxymethyl)-3,5-dihydroxy-2,2-dimethyl-3,4-dihydro-2H,6H-benzo[1,2-b-5,4-b'] dipyran-6-one	$C_{15}H_{16}O_6$	-8.68	275.0941、245.0452、221.0458、	SR	Chromone		+	+
P39	41.325	291.125	291.1227	5-O- methylvisamminol	$C_{16}H_{18}O_5$	-7.93	273.1048、233.086、205.0503	SR	Chromone			+
P40	43.12	271.0586	271.0599	Apigenin	$C_{15}H_{10}O_5$	-1.7	253.0526, 243.0656	AST	Flavonoids		+	
P41	43.813	285.0762	285.0757	Wogonin	$C_{16}H_{12}O_5$	-6.98	271.0588、270.0546	SR	Chromone		+	
P42	44.125	439.1642	439.1599	Sec-O-glucosylhamaudol	$C_{21}H_{26}O_{10}$	-9.87	424.1868、397.1327、379.1173	SR	Chromone		+	
P43	44.314	439.1616	439.1599	Cnidimoside A	$C_{21}H_{26}O_{10}$	-3.94	205.0504、259.0968、277.1065	SR	Chromone	+		
P44	43.888	285.0785	285.0757	Calycosin	$C_{16}H_{12}O_5$	-9.68	253.0510、229.0871、225.0560	AST	Flavonoids			+
P45	43.969	303.123	303.1227	Isomucronulatol	$C_{17}H_{18}O_5$	-0.99	167.0714、123.0451、106.0419、95.0489	AST	Flavonoids	+	+	
P47	45.458	517.1314	517.1341	Formononetin-7-O-Glc-6"-O-malonate	$C_{25}H_{24}O_{12}$	5.14	254.0561、137.0528	AST	Amino acids		+	
P50	47.515	947.528	947.521	Astragaloside VI	$C_{47}H_{78}O_{19}$	-7.39	431.2854、312.2325、294.2202	AST	Saponins			+

P52	43.486	287.0914	287.0914	Melilotocarpan B	$C_{16}H_{14}O_5$	-1.4	255.0655、177.0543、153.0544、138.0297	AST	Flavonoids			+
P54	49.451	389.2574	389.2534	Norcaperatic acid	$C_{20}H_{36}O_7$	-10.35	232.1715、70.665	AMR	Amino acids		+	+
P56	54.939	301.1098	301.1071	Methylnissolin	$C_{17}H_{16}O_5$	-9.16	241.0886、191.0720、167.0713	AST	Flavonoids		+	
P61	53.205	277.1095	277.1071	Hamaudol	$C_{15}H_{16}O_5$	-8.87	259.0988、244.0732、205.0506	SR	Chromone		+	+
P62	52.467	807.4530 (+Na）	807.4501	Astragaloside IV	$C_{41}H_{68}O_{14}$	-3.66	455.3528、437.3204	AST	Saponins		+	+
P63	54.344	299.0941	299.0914	Afrormosin	$C_{17}H_{14}O_5$	-9.06	284.0706、256.0725、166.0263	AST	Flavonoids		+	+
P64	53.875	269.083	269.0808	Formononetin	$C_{16}H_{12}O_4$	-8.07	254.0576、237.0587、213.0933	AST	Flavonoids	+	+	+
P65	54.679	375.1079	375.1085	Skullcapflavone II	$C_{19}H_{18}O_8$	3.2	360.0854, 345.0626, 327.0571	AST	Flavonoids			+
P66	55.417	849.4648(+Na）	849.4607	Isoastragaloside II	$C_{43}H_{70}O_{15}$	-4.97	789.4391	AST	Saponins			+
P67	56.614	645.3936(+Na）	645.3973	Astramembrannin II	$C_{35}H_{58}O_9$	5.95	437.3210	AST	Saponins	+	+	+
P70	61.144	891.4781	891.4713 (+Na）	Isoastragaloside I	$C_{45}H_{72}O_{16}$	-7.88	591.3734、380.1688	AST	Saponins			+
P72	62.32	249.1509	249.1485	Atractylenolide III	$C_{15}H_{20}O_3$	9.59	189.0945、231.1398	AMR	Lactone	+		+
P74	62.119	231.14	231.138	Atractylenolide I	$C_{15}H_{18}O_2$	-8.88	189.0845、163.0746、105.0718	AMR	Lactone	+	+	+
P76	62.177	249.1483	249.1485	6-hydroxy atractylenolide I	$C_{15}H_{20}O_3$	0.89	231.1386、213.1282、203.1446	AMR	Lactone	+	+	

P77	63.795	230.156	230.1539	atractylenolactam	$C_{15}H_{19}NO$	-8.99	214.1236、200.1069、174.092	AMR	Lactone		+	+
P83	70.163	233.1533	233.1536	Atractylenolide II	$C_{15}H_{20}O_2$	1.32	133.0869、159.0818、177.0927、187.1532、215.1435	AMR	Lactone	+		+
P85	69.821	233.1543	233.1536	3β-hydroxyatractylone	$C_{15}H_{20}O_2$	-2.99	187.1088、172.0883	AMR	Sesquiterpene		+	
P87	75.693	203.1806	203.1794	Aromadendrene, dehydro-	$C_{15}H_{22}$	-5.8	105.0702	AMR	Sesquiterpene			+
P89	76.565	253.1198	253.1183	Ser-Phe	$C_{12}H_{16}N_2O_4$	-6.02	207.1177	AST	Others			+
P96	80.148	449.1607 (+Na）	449.1571 (+Na）	Anomalin	$C_{24}H_{26}O_7$	-8.51	349.1048、327.1244	SR	Coumarin			+

Table S6. **Mass spectrometric information of metabolites components in vivo of YPFS by HPLC-Q-TOF-MS/MS.**

NO.	t_R (min)	Prototype constituents	exp	pre	**Mass Error (ppm)**	Formula	Fragment ions	Transformations	Plasma	Urine	Feces
M1	34.228	Formononetin	285.0785	285.0757	-9.68	$C_{16}H_{12}O_5$	270.0550, 225.0551	Oxidation	+	+	
M2	34.085	Formononetin	463.1253	463.1235	-3.92	$C_{22}H_{22}O_{11}$	269.0806, 245.0807, 227.0700, 137.02	Hydration, Glucuronidation	+	+	
M3	41.162	Formononetin	445.1139	445.1129	-2.20	$C_{22}H_{20}O_{10}$	269.0809, 254.0562, 213.0911	Glucuronidation	+	+	+
M4	40.47	Formononetin	431.1378	431.1337	-9.63	$C_{21}H_{18}O_{10}$	269.0827, 254.0573, 237.0558	Demethylation, Glucuronidation	+	+	+
M5	32.955	Formononetin	431.1015	431.0973	-9.83	$C_{22}H_{22}O_9$	255.0668, 113.0227	Glucoside Conjugation ($C_6H_{10}O_5$)	+	+	
M6	34.42	Formononetin	461.1082	461.1078	-0.79	$C_{22}H_{20}O_{11}$	285.0757, 253.0537, 225.0591	Oxidation, Glucuronidation	+	+	+
M7	40.126	Formononetin	301.0734	301.0707	-9.12	$C_{16}H_{12}O_6$	301.0735, 153.0155, 241.0491, 269.0461	Di-Oxidation		+	
M8	12.52	Formononetin	257.083	257.0808	-8.45	$C_{15}H_{12}O_4$	239.0698, 211.0754, 147.0440, 137.0233	Demethylation, Hydrogenation		+	
M9	44.014	Formononetin	301.1099	301.1071	-9.50	$C_{17}H_{16}O_5$	269.0812, 167.0712	Hydration, Methylation	+	+	+
M10	33.759	Formononetin	281.0931	281.0914	-5.94	$C_{17}H_{12}O_4$	268.1898, 237.1557, 219.1832, 153.0546, 137.0593, 107.0856,97.0652	Dehydrogenation, Methylation	+	+	
M11	43.911	Formononetin	303.1232	303.1227	-1.65	$C_{17}H_{18}O_5$	181.0837, 152.0461	Hydration, Reduction, Methylation	+	+	
M12	35.51	Calycosin	271.0619	271.0601	-6.47	$C_{15}H_{10}O_5$	254.1888, 186.1501, 137.0244, 60.0570	Demethylation	+	+	+
M13	53.875	Calycosin	269.0830	269.0808	-8.07	$C_{16}H_{12}O_4$	254.0576、237.0587、213.0933	Dehydroxylation (Formononetin)		+	+

M14	30.473	Calycosin	447.0955	447.092	-7.42	$C_{21}H_{18}O_{11}$	355.0613, 271.0617, 225.0702, 85.0278	Glucuronidation of M12	+	+	
M15	28.933	Calycosin	351.018	351.0169	-3.10	$C_{15}H_{10}O_8S$	271.0605, 253.0504, 225.0559, 215.0715, 137.0236	Suflation of M12	+		
M16	41.132	Calycosin	255.0676	255.0652	-9.50	$C_{15}H_{10}O_4$	237.0757, 227.0723, 199.0764	Demethylation, Dehydroxylation	+	+	+
M17	28.464	Calycosin	431.1015	431.0973	-9.83	$C_{21}H_{18}O_{10}$	428.6252, 417.2110, 414.1900, 253.0666, 73.0303	C7-O-Glucuronidation of M16	+		
M18	54.336	Calycosin	299.0936	299.0914	-7.38	$C_{17}H_{14}O_5$	239.0691, 197.0655, 95.0116	C3'-O-methylation	+	+	+
M19	41.4	Calycosin	475.123	475.1235	1.03	$C_{23}H_{22}O_{11}$	299.0914, 271.0592, 253.0488, 225.0553	C7-O Glucuronidation of M18		+	+
M20	35.133	Calycosin	365.0333	365.0326	-2.02	$C_{16}H_{12}O_8S$	285.0755, 270.0515, 253.0498, 225.0544, 173.1325, 123.080	Sulfation	+	+	
M21	22.642	Calycosin	541.0647	541.0647	-0.09	$C_{22}H_{20}O_{14}S$	541.0643, 365.0324, 270.0523	Sulfation, Glucuronidation	+	+	+
M22	34.42	Calycosin	461.1082	461.1078	-0.79	$C_{22}H_{20}O_{11}$	378.8942, 285.0753, 225.0580, 85.0285	C7-O-Glucuronidation	+	+	
M23	31.279	Calycosin	335.0248	335.022	-8.38	$C_{15}H_{10}O_7S$	255.0664, 237.0557, 227.0704, 137.0239	Demethylation, Sulfation, Dehydroxylation		+	+
M24	40.126	Calycosin	301.0734	301.0707	-9.12	$C_{16}H_{12}O_6$	301.0735, 286.0497, 153.0155, 269.0461, 134.0382	Hydroxylation	+	+	+
M25	22.633	Calycosin-7-O-glucoside	623.1655	623.1607	-7.78	$C_{28}H_{30}O_{16}$	447.1284、285.0751	C3'-O-Glucuronidation	+	+	
M26	44.081	Calycosin-7-O-glucoside	477.1436	477.1439	-9.37	$C_{23}H_{24}O_{11}$	477.1404、301.1097、253.0535、167.0715	Hydroxylation, Methylation	+	+	+

M27	26.185	Calycosin-7-O-glucoside	433.1154	433.1129	-5.73	$C_{21}H_{20}O_{10}$	257.0806, 242.0570, 163.0388, 137.023	Demethylation		+	
M28	38.183	Calycosin-7-O-glucoside	317.1045	317.1035	-8.02	$C_{17}H_{16}O_6$	299.0909, 285.0664, 257.0787, 243.1010, 163.0388, 123.04	Deglycosylation ($C_6H_{10}O_5$), Hydration, Methylation		+	
M29	35.368	Calycosin-7-O-glucoside	315.0884	315.0863	-6.64	$C_{17}H_{14}O_6$	300.0624, 283.0600, 255.0649, 243.0649, 163.0753, 123.0440	Dehydrogenation of M28		+	
M3	41.162	Ononin	445.1139	445.1129	-2.20	$C_{22}H_{20}O_{10}$	269.0809, 254.0562, 213.0911	Hydroxylation, Dehydration	+	+	+
M30	28.338	Ononin	447.1325	447.1286	-8.8	$C_{22}H_{22}O_{10}$	285.0772, 270.0566, 253.0491, 242.0600	Hydroxylation	+		+
M31	33.96	Ononin	461.1118	461.1078	-8.61	$C_{22}H_{20}O_{11}$	285.0776, 270.0540, 225.0566, 137.0237	Dehydrogenation, Di-Oxidation			
M32	26.185	Ononin	433.1154	433.1129	-5.73	$C_{21}H_{20}O_{10}$	257.0806, 242.0570, 163.0388, 137.023	Oxidation, Demethylation		+	
M33	43.978	Methylnissolin	477.1404	477.1391	-2.65	$C_{23}H_{24}O_{11}$	301.1074, 167.0699	Glucuronidation	+	+	
M34	34.085	Methylnissolin	461.1083	461.1078	-1.00	$C_{22}H_{20}O_{11}$	285.0764, 253.0485, 225.0558, 213.0541, 137.024	Demethylation, Dehydrogenation, Glucuronidation	+	+	
M35	40.998	Methylnissolin	269.0834	269.0808	-9.57	$C_{16}H_{12}O_4$	253.0509, 237.0578, 213.0931	Dehydration, Demethylation	+	+	+
M36	35.435	Methylnissolin	491.1223	491.1184	-7.95	$C_{23}H_{22}O_{12}$	315.0879, 85.0292, 199.0221	Dehydrogenation, Glucuronidation	+	+	+
M37	41.621	Methylnissolin	475.1224	475.1235	2.29	$C_{23}H_{22}O_{11}$	299.0913, 284.0677, 267.0654, 211.0750, 137	Dehydrogenation, Glucuronidation	+	+	+

M38	52.266	Astragaloside IV (+Na）	513.3775	513.3731	-8.97	$C_{30}H_{50}O_5$	489.3570, 445.2027	Desugaring ($-C_6H_{10}O_5$, $-C_5H_8O_4$)	+		
M39	47.038	Astragaloside IV (+Na）	511.3277	511.3262	-3.2	$C_{30}H_{48}O_5$	443.3198	Dehydrogenation of M38			+
M40	79.965	Astragaloside IV (+Na）	591.4888	591.4889	0.15	$C_{30}H_{48}O_8S$	184.0728, 342.369	Sulfation of M39	+	+	
M41	32.742	Atractylenolide III	265.1433	265.1434	0.51	$C_{15}H_{20}O_4$	247.1310, 229.1206, 211.1144, 201.1230	Hydroxylation	+	+	+
M42	20.564	Atractylenolide III	281.1411	281.1384	-9.82	$C_{15}H_{20}O_5$	227.1071, 245.1171	Hydroxylation of M41	+	+	+
M43	27.167	Atractylenolide III	263.1291	263.1278	-5.01	$C_{15}H_{18}O_4$	245.1172, 217.1222	Dehydrogenation of M41	+	+	
M44	46.695	Atractylenolide III	283.1564	283.254	-8.51	$C_{15}H_{22}O_5$	305.1347, 265.1433, 247.1337, 229.1239	Hydration, Hydroxylation	+		
M45	48.403	Atractylenolide III/ Atractylenolide I	247.1349	247.1329	-8.24	$C_{15}H_{18}O_3$	247.1346, 229.1227, 201.1316	Dehydrogenation/Oxidation		+	+
M46	40.126	Atractylenolide III	423.1691	423.165	-9.81	$C_{21}H_{26}O_9$	423.1688, 408.1291, 407.1715, 405.2176	Glucuronidation of M45	+	+	
M47	33.424	Atractylenolide III	441.1794	441.1755	-8.81	$C_{21}H_{28}O_{10}$	423.1276, 405.1532, 229.1237	Hydration of M46		+	+
M48	20.497	Atractylenolide I	281.1402	281.1384	-6.60	$C_{15}H_{20}O_5$	217.1735, 187.0978, 165.0718, 128.0613, 91.0546	Hydration, Di-Oxidation			+
M49	35.845	Atractylenolide I	281.1195	281.1206	3.90	$C_{15}H_{20}O_3S$	263.1075, 239.1078, 187.0756, 133.0650, 107.0501	Di-oxidation, Reduction, Sulfation			+

M50	19.483	Atractylenolide I	384.1507	384.1475	-8.26	$C_{18}H_{25}NO_6S$	366.1380, 338.1357, 320.1309, 295.1017	Cysteine ($C_3H_7NO_2S$) Conjugation, Di-oxidation		+	
M51	25.85	Atractylenolide I	229.124	229.1223	-7.43	$C_{15}H_{16}O_2$	229.12, 201.09, 173.10, 157.10	Dehydrogenation		+	
M52	40.202	Atractylenolide I	249.1506	249.1585	-8.38	$C_{15}H_{20}O_3$	231.1405, 213.1293, 185.1343, 157.1020	Hydration		+	+
M53	27.435	Atractylenolide I	265.1433	265.1434	0.510	$C_{15}H_{20}O_4$	172.0920, 129.0721, 91.0567	Oxidation of M52	+		
M54	19.818	Cimifugin	323.1151	323.1125	-7.98	$C_{16}H_{18}O_7$	247.0619, 259.0609, 221.0462	Hydroxylation	+	+	
M55	25.984	Cimifugin	483.1544	483.1497	-9.74	$C_{22}H_{26}O_{12}$	307.1206, 289.1094, 261.1144, 259.0625, 235.0621	Glucuronidation	+	+	+
M56	22.432	Scopoletin	369.0849	369.0816	-8.90	$C_{16}H_{16}O_{10}$	369. 0816，256. 9261，193. 0494， 133. 0283，122. 0425	Glucuronidation	+		
M57	28.204	Scopoletin	273.0664	273.0652	-6.26	$C_{10}H_8O_7S$	177.0555, 145.0284, 117.0343, 89.0394, 63.0227	Sulfation			+

Table S7. **The serial number of the 42 prototype compounds.**

NO.	NO.(Peak)	Compounds	Source
AST1	P56	Methylnissolin	AST
AST2	P52	Melilotocarpan B	AST
AST3	P63	Afrormosin	AST
AST4	P62	Astragaloside IV	AST
AST5	P50	Astragaloside VI	AST
AST6	P67	Astramembrannin II	AST
AST7	P65	Skullcapflavone II	AST
AST8	P44	Calycosin	AST
AST9	P31	Calycosin-7-O-Glc-6"-O-malonate	AST
AST10	P24	Calycosin-7-O-glucoside	AST
AST11	P70	Isoastragaloside I	AST
AST12	P47	Formononetin-7-O-Glc-6"-O-malonate	AST
AST13	P64	Formononetin	AST
AST14	P66	Isoastragaloside II	AST
AST15	P45	Isomucronulatol	AST
AST16	P40	Apigenin	AST
AST17	P35	Ononin	AST
AST18	P30	Pratensein-7-O-glucoside	AST
AST19	P89	Ser-Phe	AST
AST20	P7	Adenosine	AST
SR1	P38	3,4-dihydro-2H,6H-benzo[1,2-b-5,4-b'] dipyran-6-one	SR
SR2	P39	5-O- methylvisamminol	SR
SR3	P25	5-O-methylvisamminoside	SR
SR4	P96	Anomalin	SR
SR5	P27	Cimifugin	SR
SR6	P43	Cnidimoside A	SR
SR7	P18	Divaricatacid	SR
SR8	P61	Hamaudol	SR
SR9	P20	Prim-O-glucosylcimifugin	SR
SR10	P21	Scopoletin	SR
SR11	P42	Sec-O-glucosylhamaudol	SR
SR12	P15	Undulatoside A	SR
SR13	P41	Wogonin	SR
AMR1	P85	3β-hydroxyatractylone	AMR
AMR2	P76	6-hydroxy atractylenolide I	AMR
AMR3	P87	Aromadendrene, dehydro-	AMR
AMR4	P77	atractylenolactam	AMR
AMR5	P72	Atractylenolide III	AMR
AMR6	P74	Atractylenolide I	AMR
AMR7	P83	Atractylenolide II	AMR
AMR8	P28	Atractylone	AMR
AMR9	P54	Norcaperatic acid	AMR

Table S8. **The information from network topology analysis on the 42 compounds.**

Name	BetweennessCentrality	ClosenessCentrality	Degree
SR5	0.04582	0.43532	53
SR13	0.04964	0.42147	45
AST13	0.01492	0.40228	34
AST7	0.01131	0.40076	33
AMR5	0.01635	0.39626	30
AST11	0.00927	0.39479	29
AST2	0.00953	0.39332	28
AST3	0.0156	0.39042	27
SR4	0.00977	0.38899	24
AST1	0.00469	0.38757	24
AST6	0.00571	0.38616	24
AST8	0.00502	0.38757	24
AST16	0.00469	0.38757	24
AST15	0.00513	0.38757	23
AMR6	0.00866	0.38475	23
AMR9	0.026875	0.384755	22
AST19	0.006733	0.381982	20
AST4	0.004015	0.38061	20
AST5	0.00461	0.381982	20
AST9	0.003276	0.38061	20
AST14	0.004354	0.381982	20
AST17	0.002995	0.38061	20
AMR7	0.005281	0.379249	18
SR7	0.005243	0.379249	18
SR10	0.004367	0.379249	18
AST10	0.001675	0.376554	17
AMR2	0.00472	0.376554	16
SR6	0.002567	0.377897	16
SR11	0.003703	0.373898	16
AST12	0.00209	0.373898	16
AST18	0.001772	0.373898	16
AMR8	0.012344	0.368696	15
SR2	0.002544	0.373898	14
SR12	0.001556	0.368696	13
AMR3	0.003189	0.358714	12
AST20	0.002908	0.366149	12
AMR4	0.001908	0.363636	11
SR8	0.001504	0.369983	10
SR9	9.67E-04	0.363636	9
SR3	0.001053	0.362393	8
SR1	5.88E-04	0.338118	6
AMR1	4.92E-04	0.361158	5

Table S9. The degree information from PPI on the top 20 core targets.

Shared name	degree.
TNF	108
IL6	107
ALB	102
IL1B	91
AKT1	82
VEGFA	76
CXCL8	75
STAT3	73
EGFR	71
TLR4	71
IL4	70
CCL2	69
MMP9	69
JUN	66
PTGS2	64
FN1	63
PPARG	62
IL2	60
HIF1A	57
EDN1	53

Table S10. The detailed information on the top 30 GO terms.

Category	Description	Serial number	Count
GO Biological Processes	response to xenobiotic stimulus	GO:0009410	48
GO Biological Processes	inflammatory response	GO:0006954	48
GO Biological Processes	blood circulation	GO:0008015	42
GO Biological Processes	positive regulation of cell motility	GO:2000147	47
GO Biological Processes	regulation of hormone levels	GO:0010817	43
GO Biological Processes	response to hormone	GO:0009725	46
GO Biological Processes	cellular response to lipid	GO:0071396	39
GO Biological Processes	positive regulation of phosphorylation	GO:0042327	44
GO Biological Processes	cell activation	GO:0001775	42
GO Biological Processes	regulation of secretion	GO:0051046	38
GO Cellular Components	membrane raft	GO:0045121	21
GO Cellular Components	vesicle lumen	GO:0031983	20
GO Cellular Components	receptor complex	GO:0043235	20
GO Cellular Components	endocytic vesicle	GO:0030139	15
GO Cellular Components	presynaptic membrane	GO:0042734	10
GO Cellular Components	external side of plasma membrane	GO:0009897	14
GO Cellular Components	apical plasma membrane	GO:0016324	13
GO Cellular Components	early endosome	GO:0005769	13
GO Cellular Components	cell projection membrane	GO:0031253	11
GO Cellular Components	basal part of cell	GO:0045178	10
GO Molecular Functions	G protein-coupled amine receptor activity	GO:0008227	15
GO Molecular Functions	heme binding	GO:0020037	21
GO Molecular Functions	cytokine receptor binding	GO:0005126	24
GO Molecular Functions	steroid binding	GO:0005496	15
GO Molecular Functions	G protein-coupled receptor binding	GO:0001664	16
GO Molecular Functions	G protein-coupled neurotransmitter receptor activity	GO:0099528	6
GO Molecular Functions	protein homodimerization activity	GO:0042803	22
GO Molecular Functions	protein kinase activity	GO:0004672	19
GO Molecular Functions	phosphatase binding	GO:0019902	12
GO Molecular Functions	kinase binding	GO:0019900	21

Table S11. The details on the top 20 KEGG pathways.

pathway	enrichment	pvalue	count
hsa05200: Pathways in cancer	12.65561	2.21E-13	39
hsa04151: PI3K-Akt signaling pathway	11.01888	9.57E-12	30
hsa04080: Neuroactive ligand-receptor interaction	9.905333	1.24E-10	29
hsa04933: AGE-RAGE signaling pathway in diabetic complications	19.94278	1.14E-20	25
hsa05171: Coronavirus disease - COVID-19	11.26467	5.44E-12	25
hsa04064: NF-kappa B signaling pathway	11.23314	5.85E-12	24
hsa04020: Calcium signaling pathway	10.1191	7.6E-11	24
hsa04620: Toll-like receptor signaling pathway	9.181115	6.59E-10	22
hsa04010: MAPK signaling pathway	6.323045	4.75E-07	21
hsa04060: Cytokine-cytokine receptor interaction	6.299465	5.02E-07	21
hsa04659: Th17 cell differentiation	13.14413	7.18E-14	20
hsa04066: HIF-1 signaling pathway	13.06773	8.56E-14	20
hsa04024: cAMP signaling pathway	6.173065	6.71E-07	18
hsa05208: Chemical carcinogenesis - reactive oxygen species	6.117921	7.62E-07	18
hsa05010: Alzheimer disease	3.111259	7.74E-04	18
hsa04668: TNF signaling pathway	9.701597	1.99E-10	17
hsa04926: Relaxin signaling pathway	8.769551	1.70E-09	17
hsa05152: Tuberculosis	6.686133	2.06E-07	17
hsa04062: Chemokine signaling pathway	6.301899	4.99E-07	17
hsa04657:IL-17 signaling pathway	8.787157	1.63E-09	15

Table S12. **Molecular docking binding energy (KJ/mol).**

Ingredients	TNF-α	IL-6	ALB	IL-1β	AKT1	VEGFA	CXCL8	STAT3	EGFR	TLR4
**AMR5**	-5.938	-3.38	-6.143	-3.861	-6.379	-5.061	-3.409	-4.57	-6.154	-4.432
**AMR6**	-4.377	-5.23	-5.303	-4.109	-6.218	-4.928	-3.829	-3.887	-6.161	-3.46
**AST1**	-5.994	-4.4	-6.574	-5.05	-8.52	-5.506	-4.202	-4.974	-7.038	-5.189
**AR11**	-0.94	-1.442	-6.899	-4.678	-7.861	-5.424	-3.869	-4.355	-6.846	-5.597
**AST13**	-5.662	-3.598	-5.887	-5.199	-6.327	-5.628	-4.07	-4.419	-3.904	-4.148
**AST15**	-6.03	-4.191	-6.066	-4.384	-7.786	-5.746	-3.73	-4.32	-6.788	-4.689
**AST16**	-3.596	-3.138	-3.944	-3.030	-5.112	-3.304	-2.521	-2.984	-4.223	-3.113
**AR2**	-5.968	-4.668	-6.784	-5.024	-8.218	-5.736	-4.294	-4.686	-6.566	-5.576
**AST3**	-2.342	-3.861	-6.632	-4.407	-7.242	-5.008	-3.662	-4.083	-6.766	-5.075
**AST6**	-5.45	-4.145	-3.621	-4.743	-7.643	-5.016	-3.948	-3.795	-5.096	-2.665
**AST7**	-6.128	-4.23	-6.363	-4.728	-6.468	-5.5	-4.058	-4.674	-6.055	-4.728
**AST8**	-2.508	-1.067	-6.624	-4.586	-7.663	-5.583	-3.619	-4.591	-7.461	-5.402
**SR13**	-6.474	-3.548	-6.008	-4.213	-7.424	-4.474	-3.765	-2.64	-7.045	-2.598
**SR4**	-4.954	-2.279	-6.137	-2.611	-7.477	-3.99	-1.653	-2.904	-4.354	-2.691
**SR5**	-5.921	-3.786	-7.422	-4.659	-7.699	-7.916	-4.302	-4.178	-7.327	-5.717

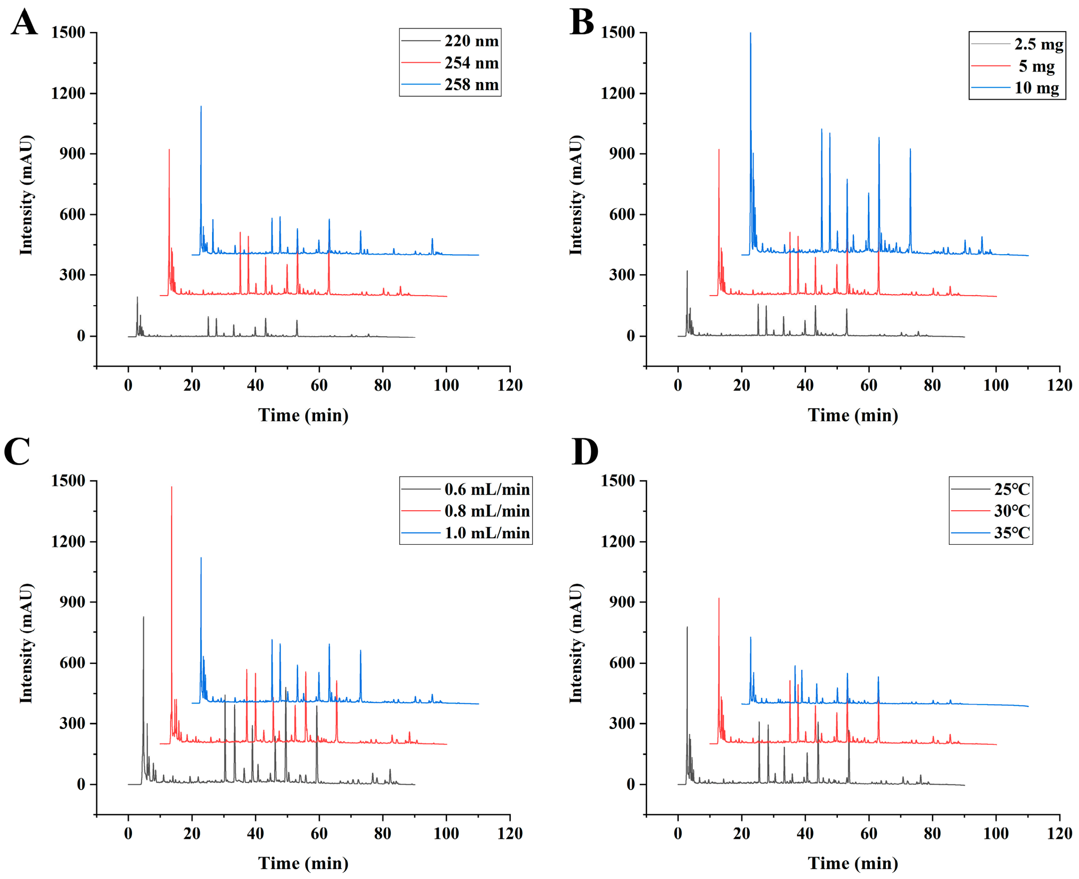

Figure S1. Optimization of chromatographic conditions. (A) Wave length; (B) Injection volume; (C) Flow rate; (D) Column temperature.

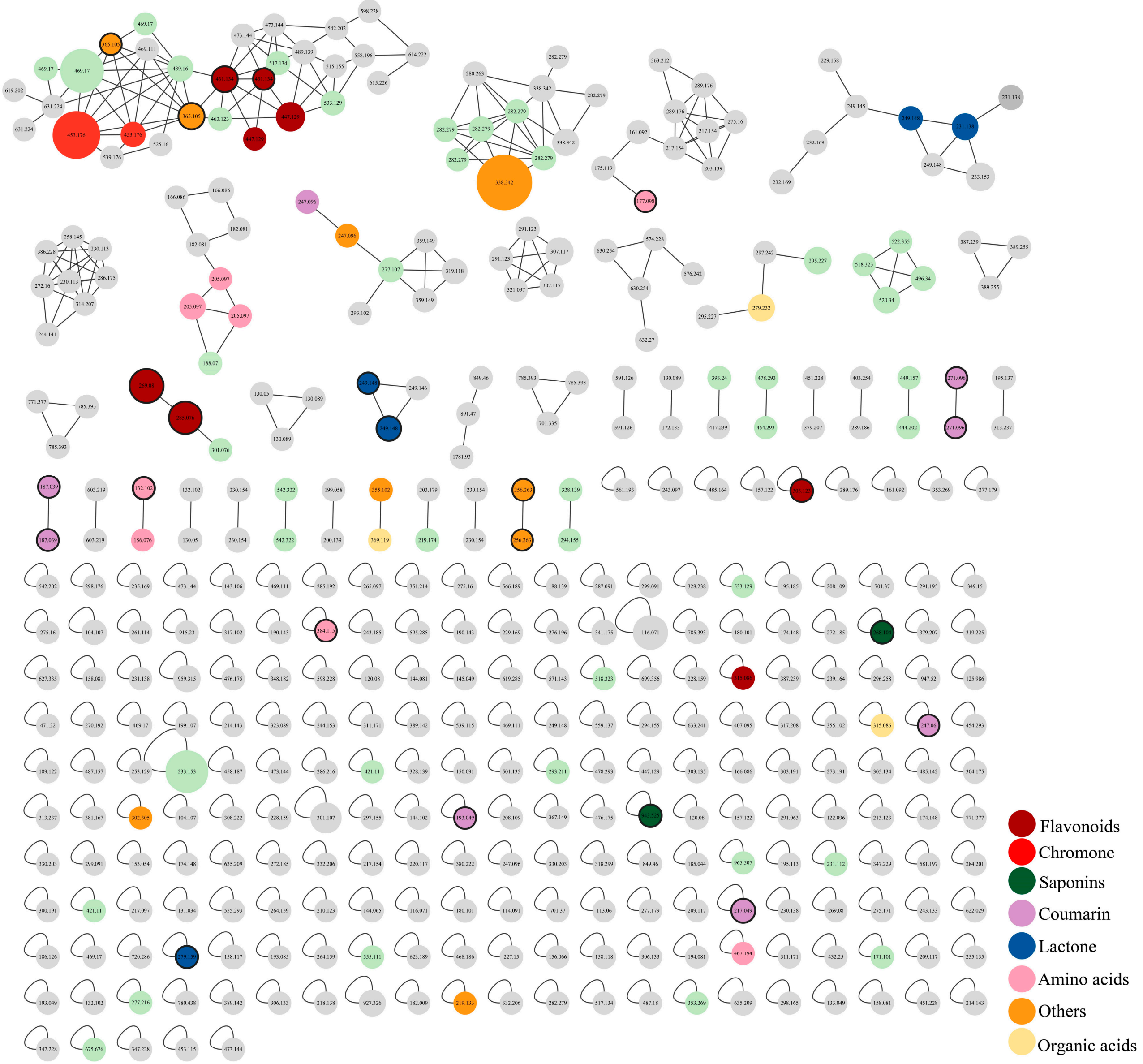

Figure S2. Molecular network of the constituents in YPFS. The node color indicated the chemical type of the compound (red: flavonoids; green: saponins; purple: coumarin; blue: lactone; pink: amino acids; orange: others; pale yellow: organic acids).

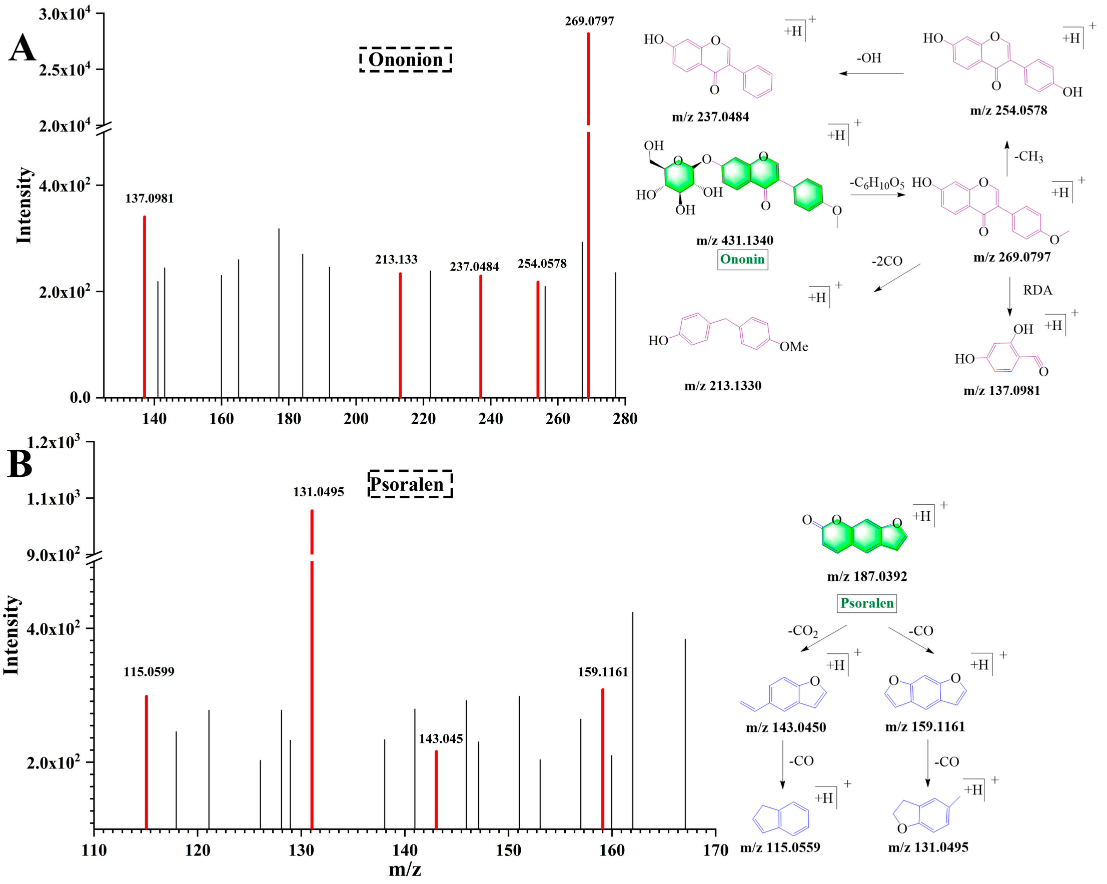

Figure S3. Chemical fragmentation pathways of chemical components in YPFS. (A) ononin; (B) psoralen.

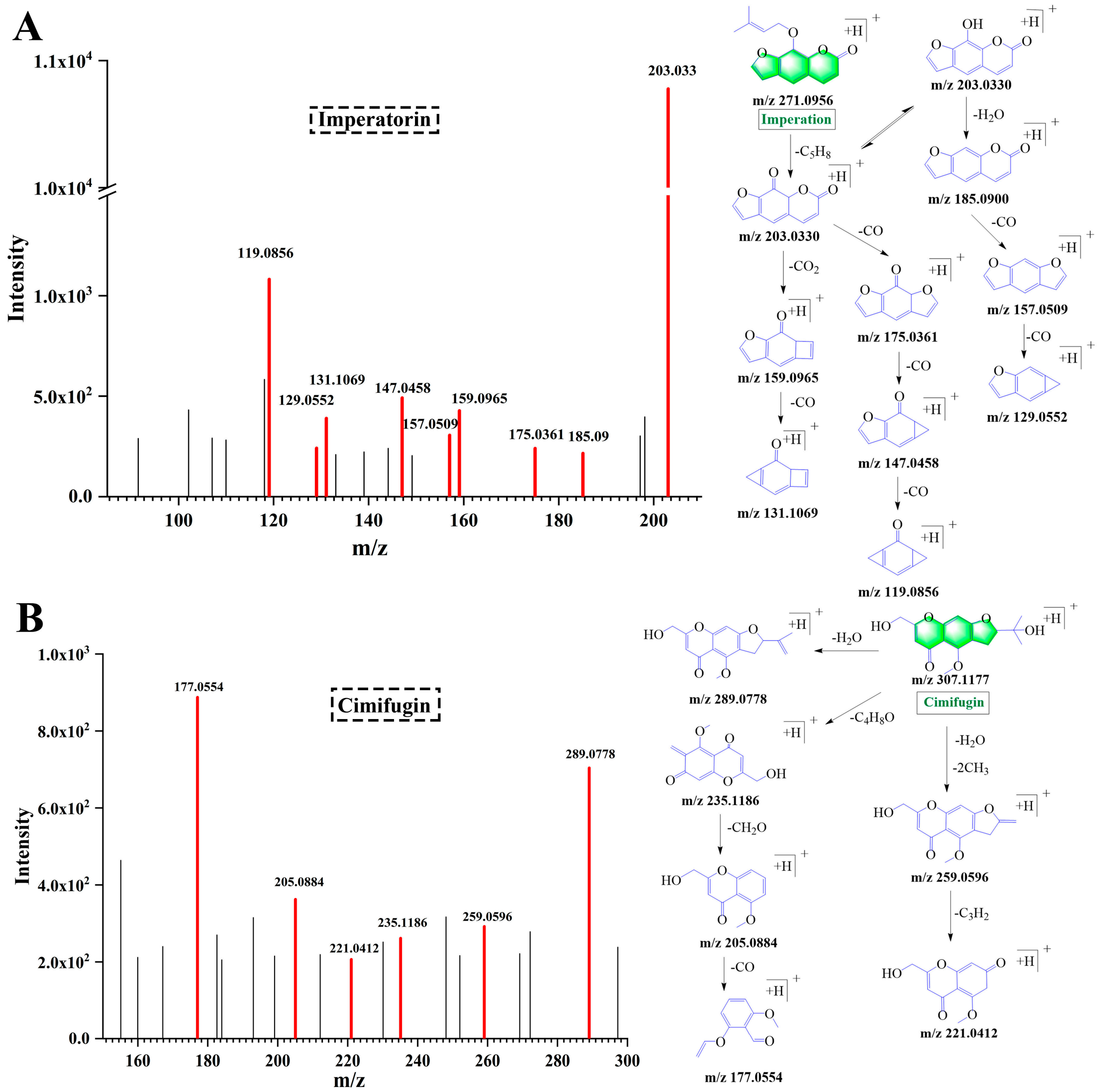

Figure S4. Chemical fragmentation pathways of chemical components in YPFS. (A) imperatorin; (B) cimifugin.

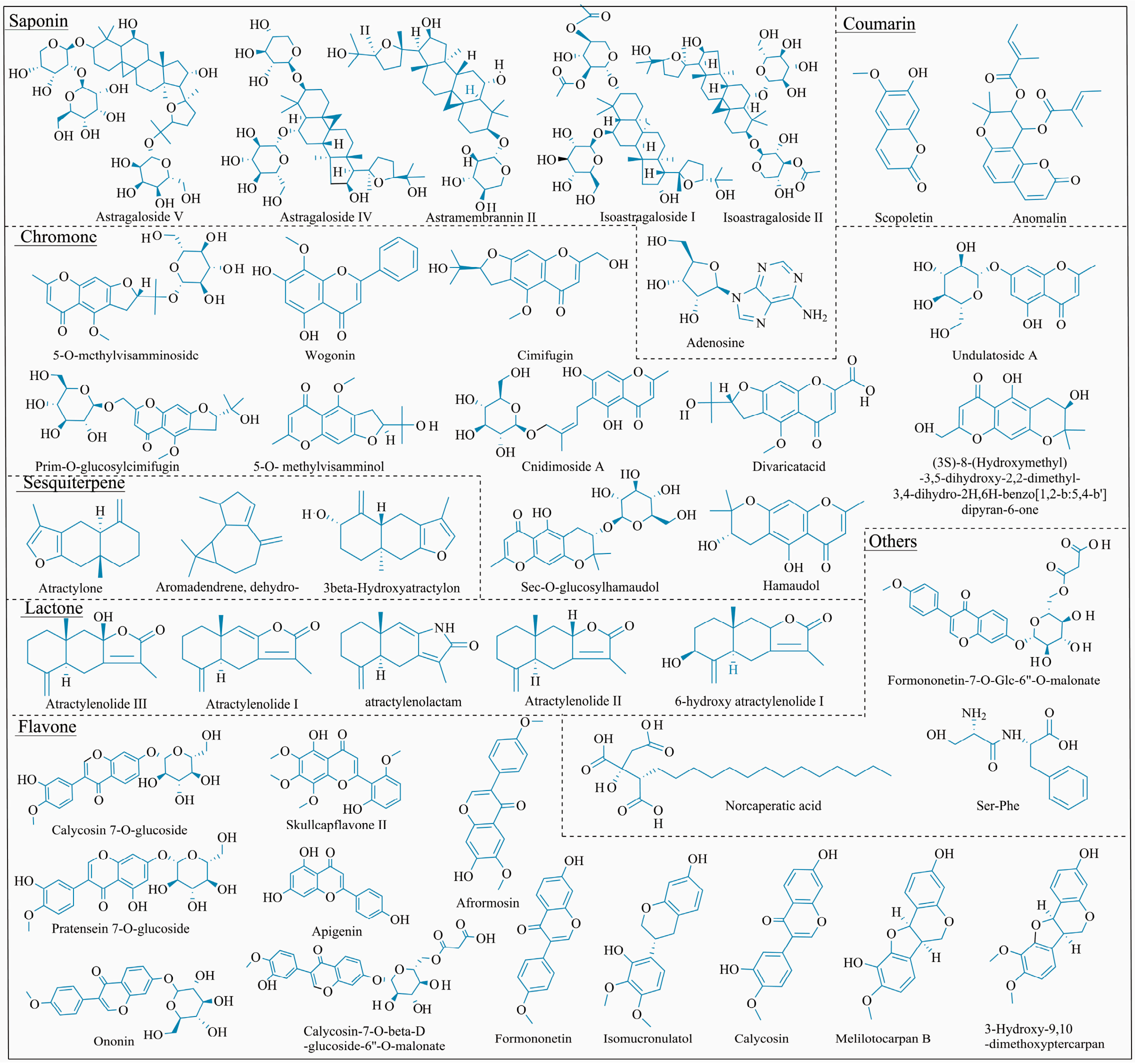

Figure S5. The structural formula of prototypical components from YPFS in AR mice.

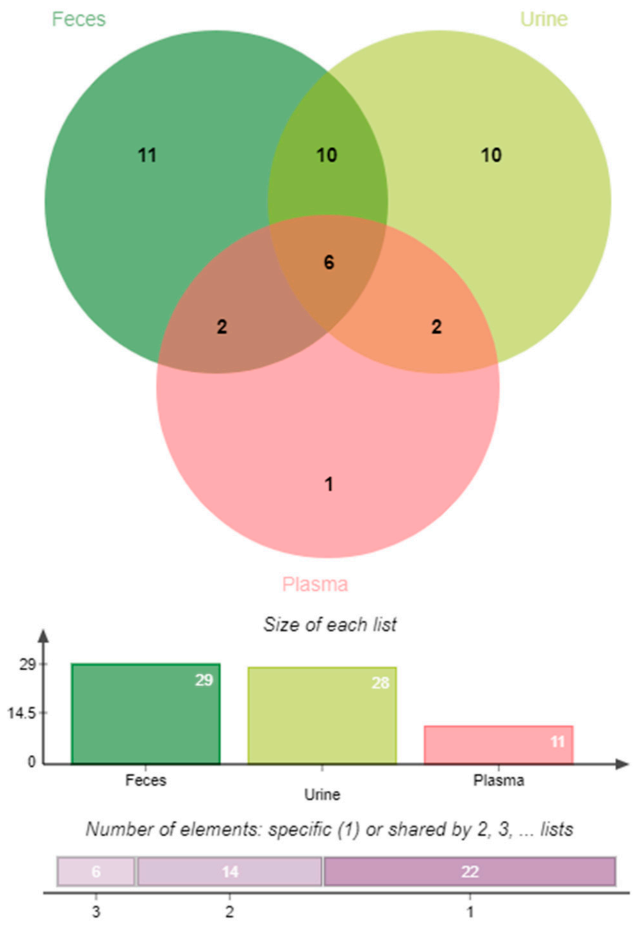

Figure S6. Venn diagram detailing the proportions of plasma, urine and feces.

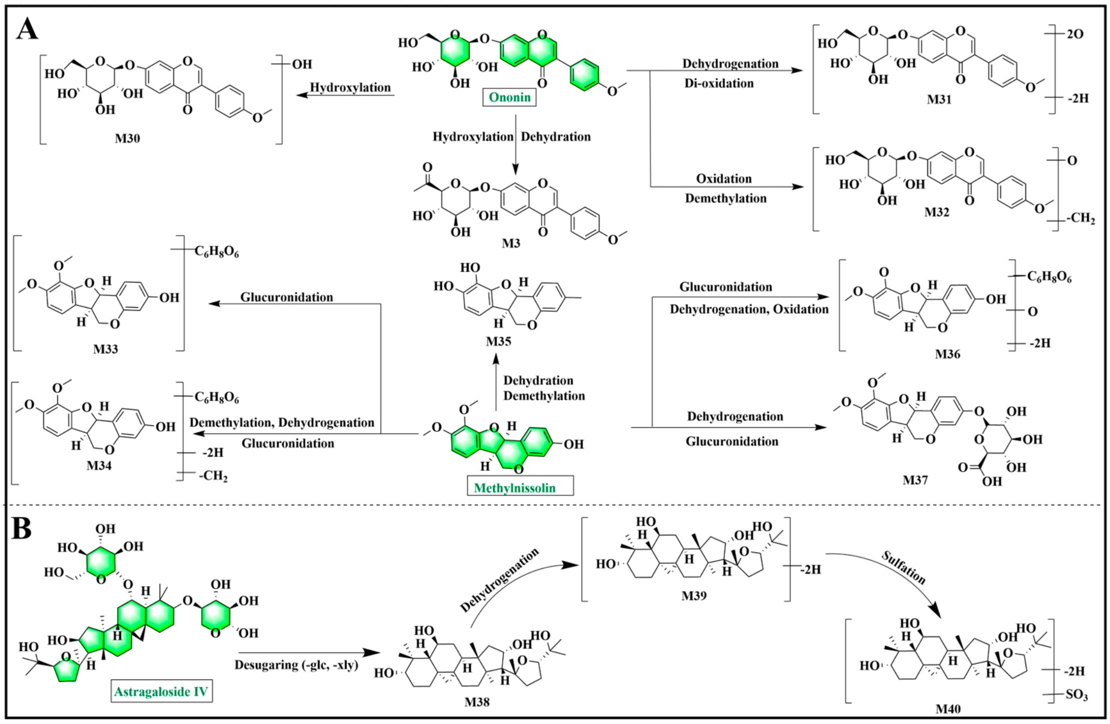

Figure S7. The possible metabolic pathways of (A) ononin and methylnissolin (B) astragaloside IV from YPFS in AR mice.

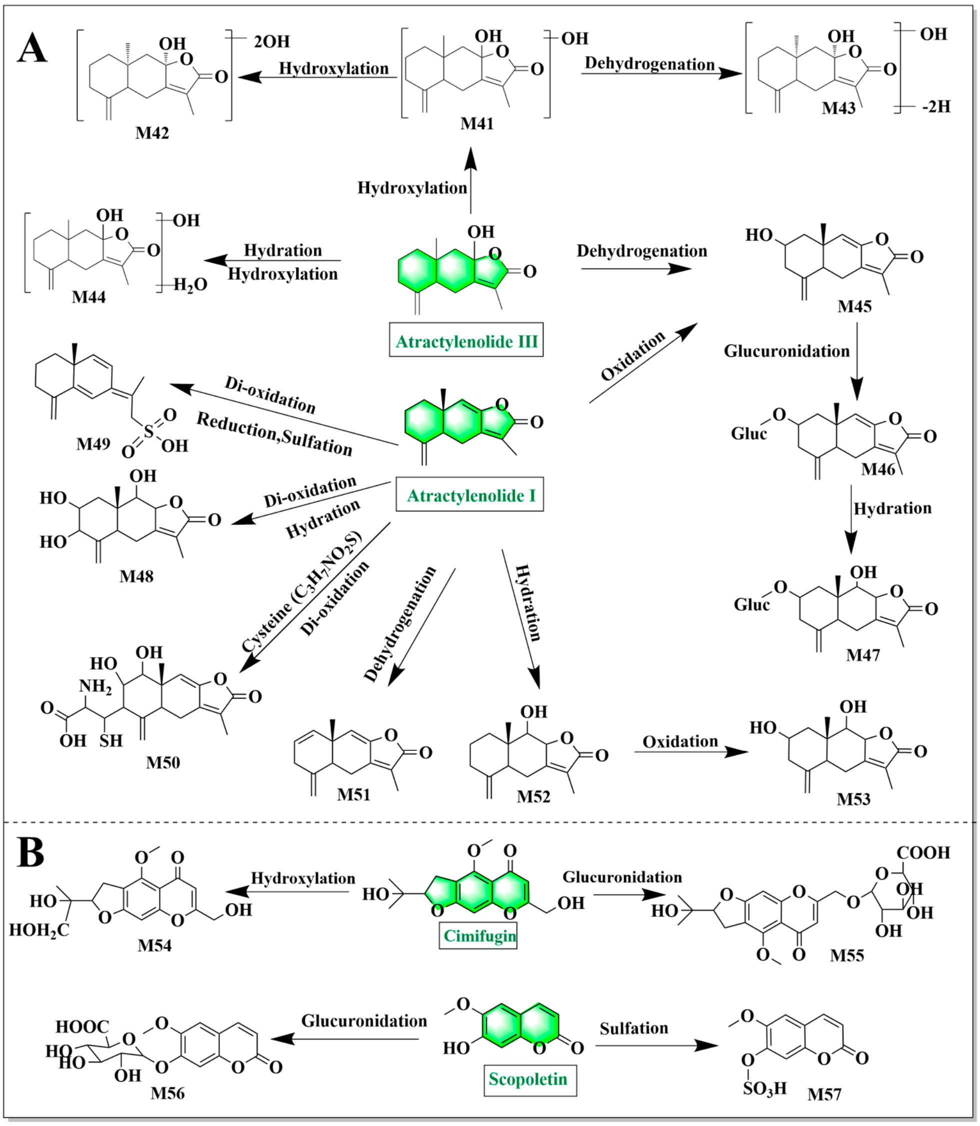

Figure S8. The possible metabolic pathways of (A) atractylenolide I and atractylenolide III (B) cimfugin and scopoletin from YPFS in AR mice.

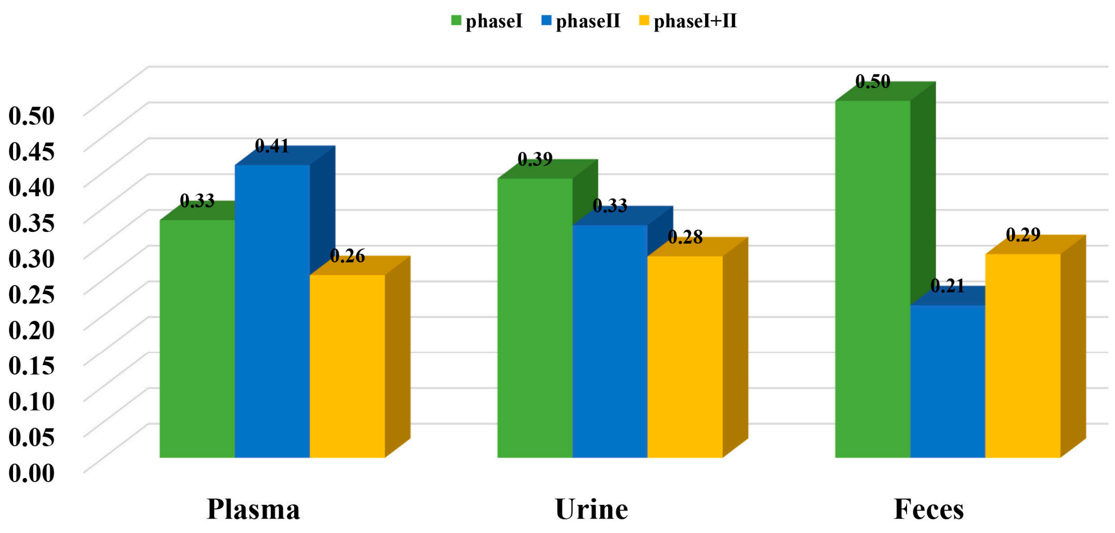

Figure S9. The proportion of metabolic types of YPFS in the plasma, urine, and feces of AR mice.

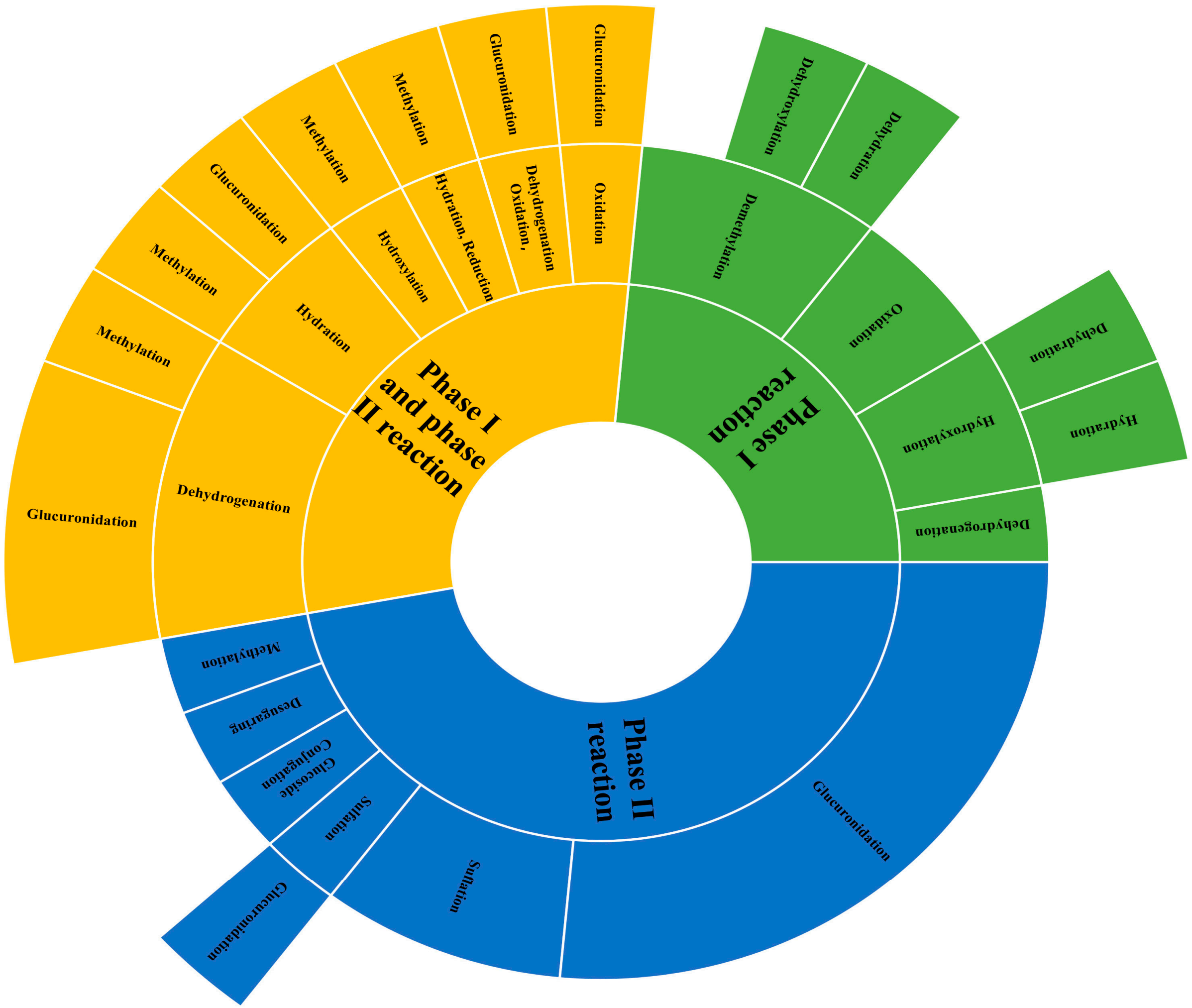

Figure S10. Metabolic types of YPFS in the plasma of AR mice.

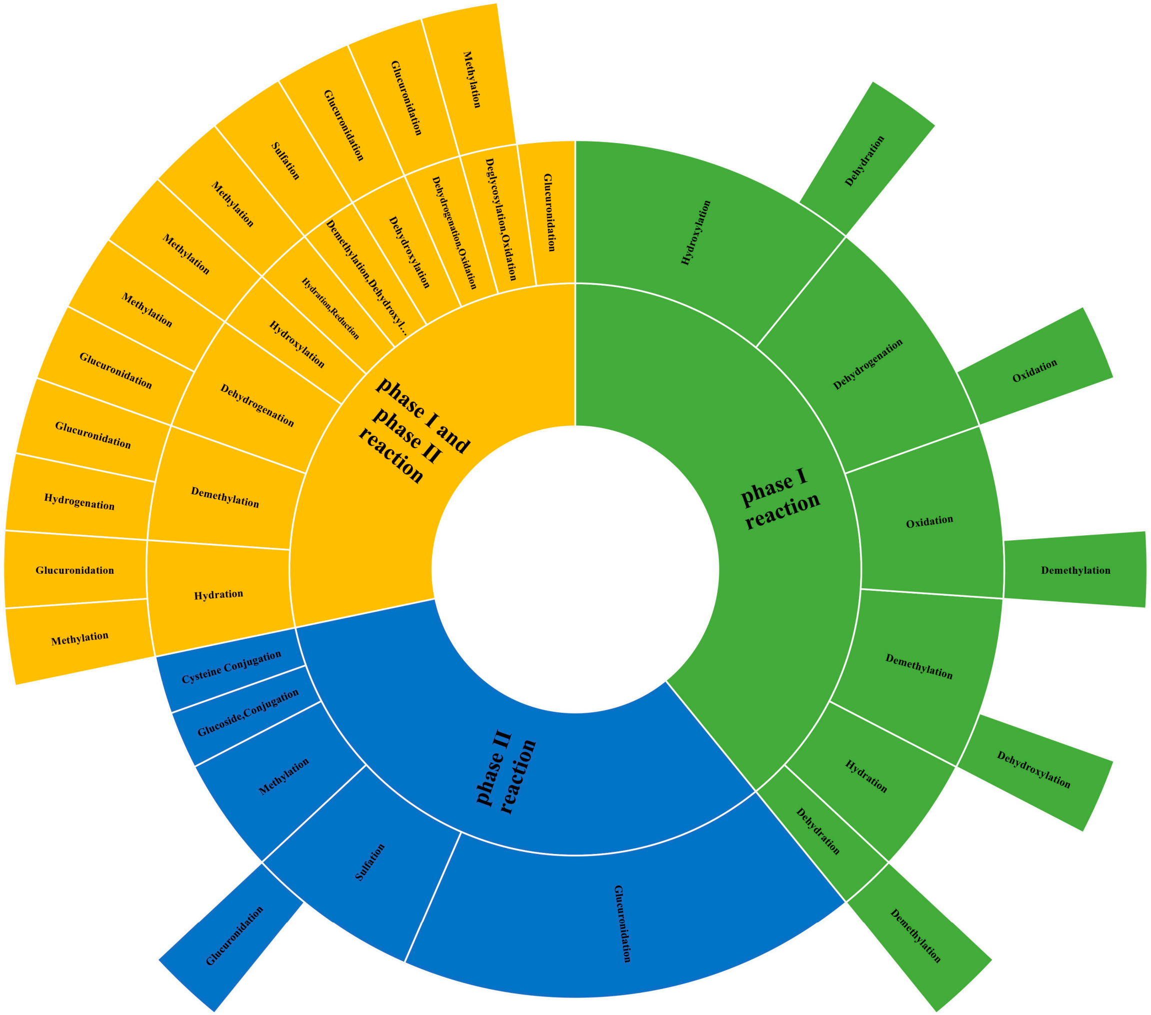

Figure S11. Metabolic types of YPFS in the urine of AR mice.

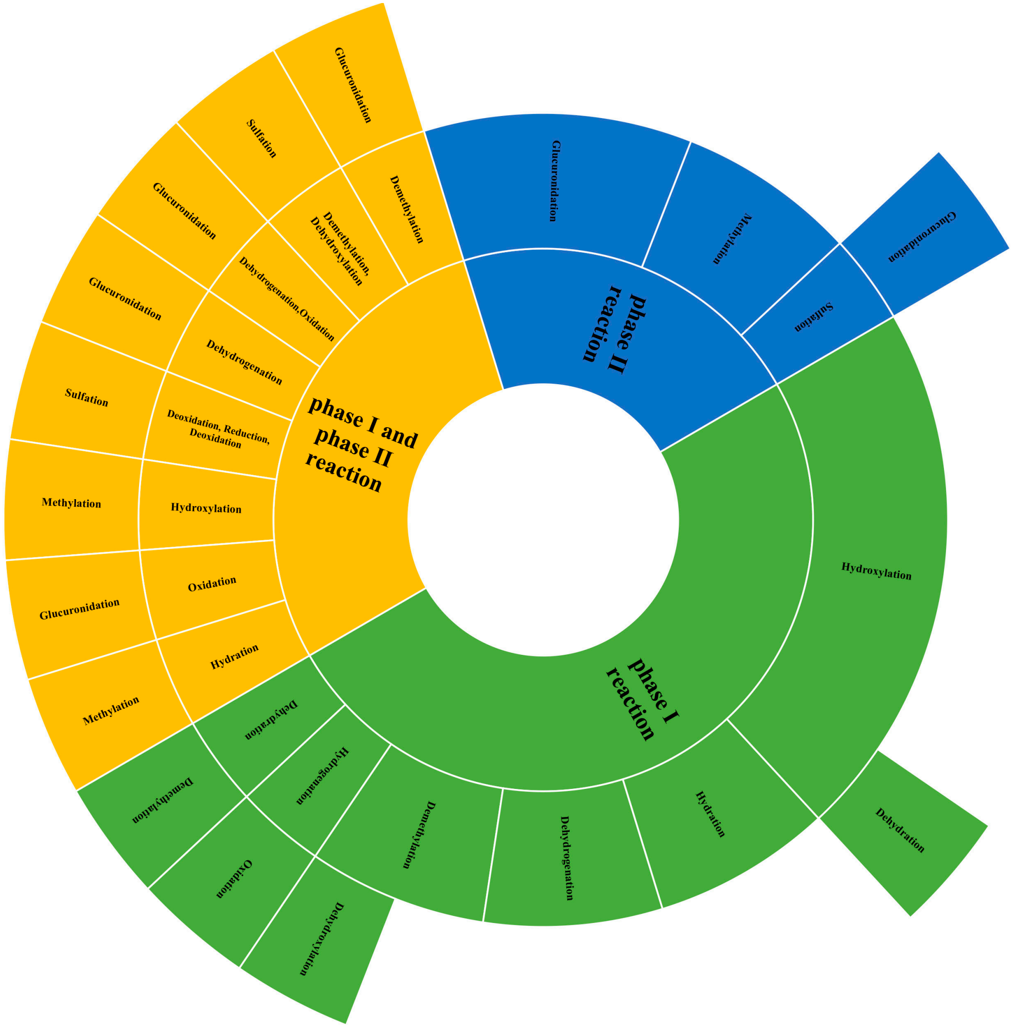

Figure S12. Metabolic types of YPFS in the feces of AR mice.

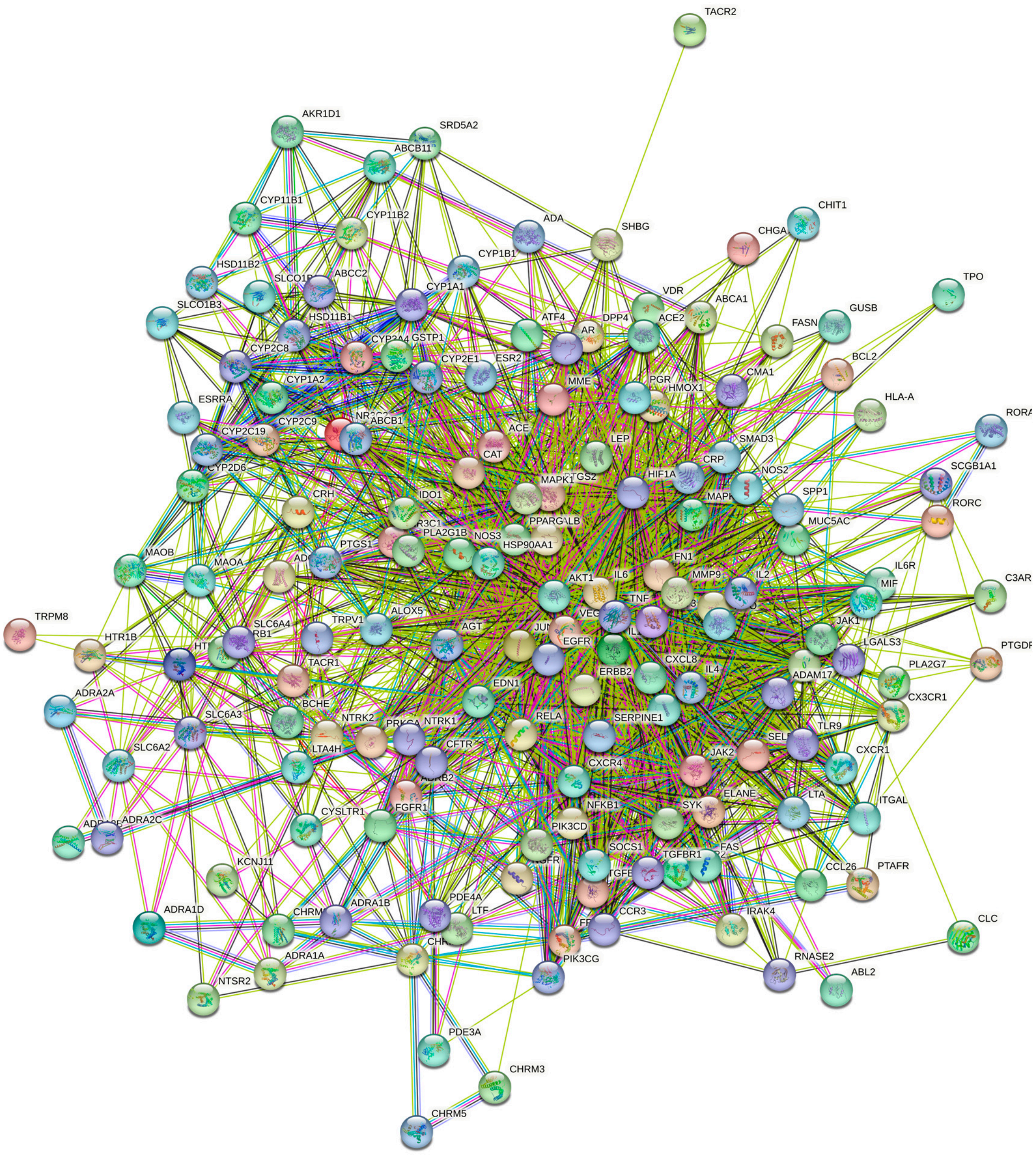

Figure S13. Protein–protein interaction (PPI) network.

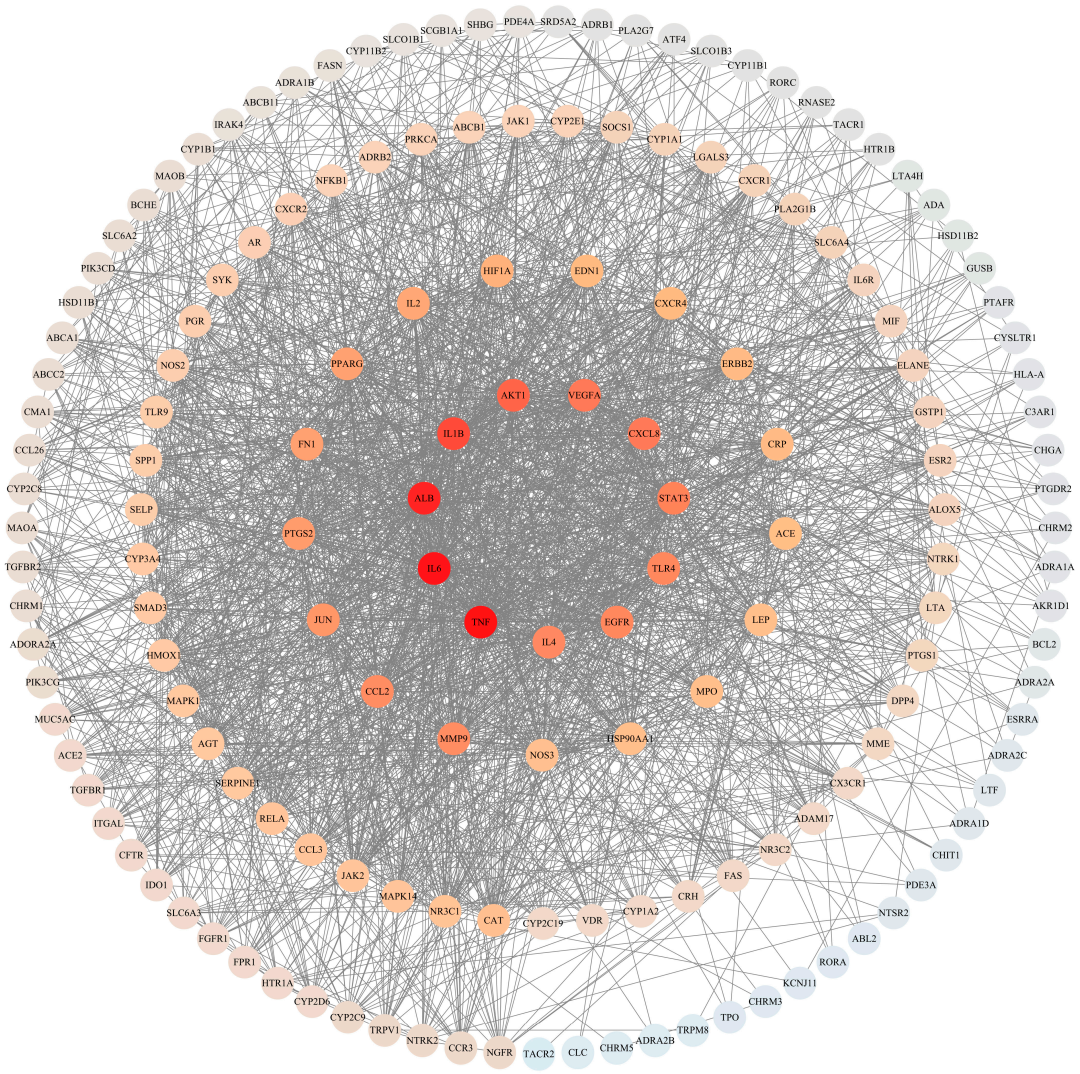

Figure S14. Circle plots for PPI network.

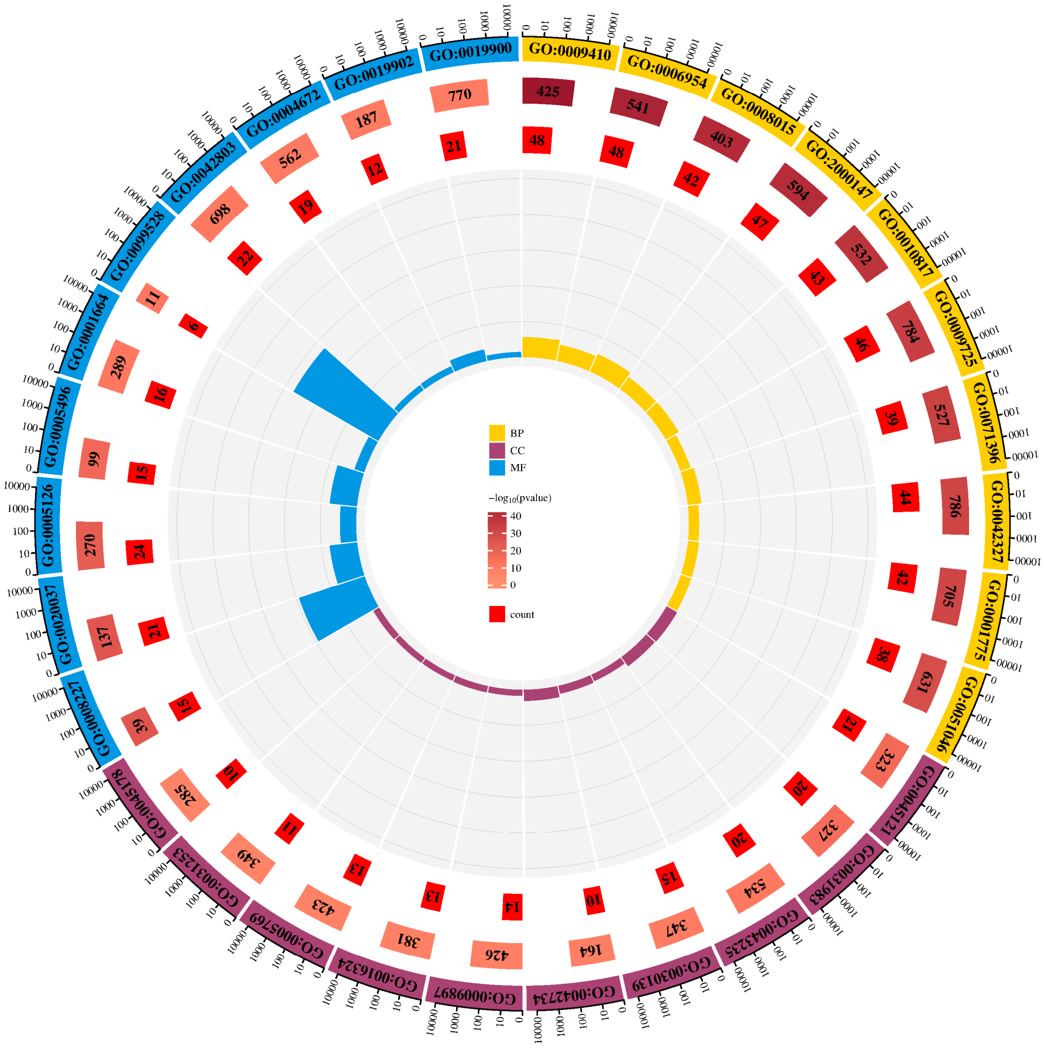

Figure S15. Circle plots for GO analysis.

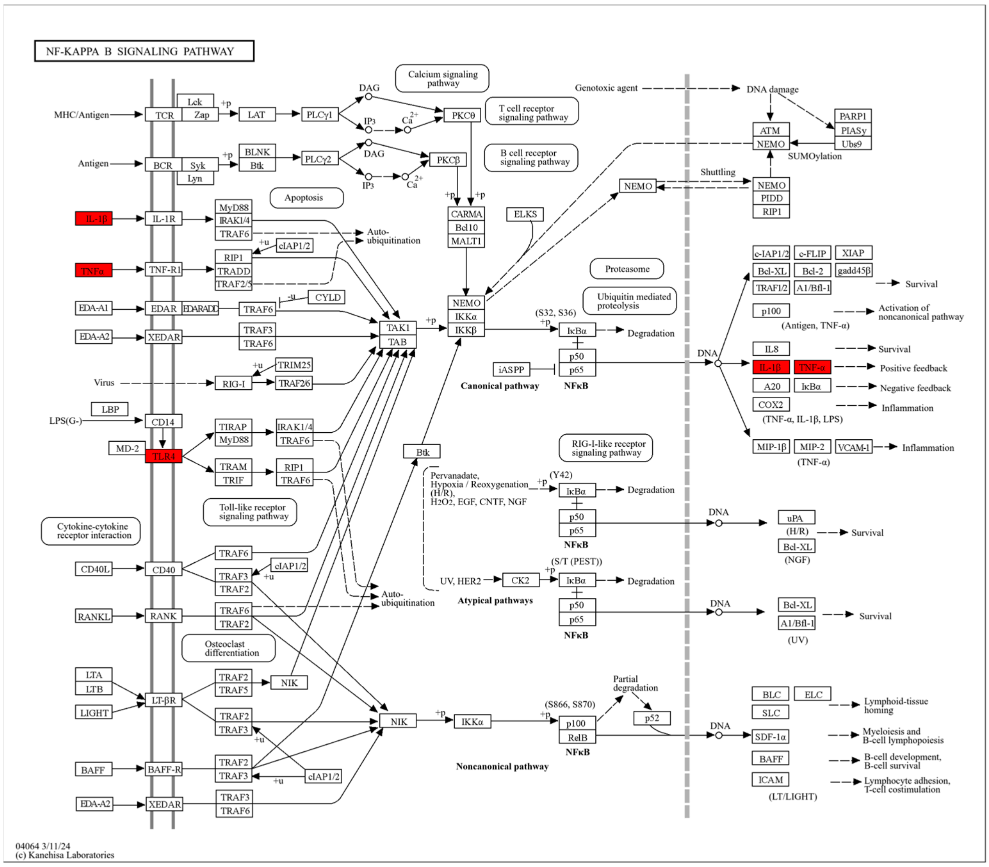

Figure S16. NF-κB pathway diagram with genes predicted to be YPF-AR targets (red).
